# High-Resolution Coproecology: Using Coprolites to Reconstruct the Habits and Habitats of New Zealand’s Extinct Upland Moa (*Megalapteryx didinus*)

**DOI:** 10.1371/journal.pone.0040025

**Published:** 2012-06-29

**Authors:** Jamie R. Wood, Janet M. Wilmshurst, Steven J. Wagstaff, Trevor H. Worthy, Nicolas J. Rawlence, Alan Cooper

**Affiliations:** 1 Landcare Research, Lincoln, Canterbury, New Zealand; 2 School of Biological, Earth and Environmental Sciences, University of New South Wales, Sydney, New South Wales, Australia; 3 Australian Centre for Ancient DNA, School of Earth and Environmental Sciences, University of Adelaide, Adelaide, South Australia, Australia; Natural History Museum of Denmark, University of Copenhagen, Denmark

## Abstract

Knowledge about the diet and ecology of extinct herbivores has important implications for understanding the evolution of plant defence structures, establishing the influences of herbivory on past plant community structure and composition, and identifying pollination and seed dispersal syndromes. The flightless ratite moa (Aves: Dinornithiformes) were New Zealand’s largest herbivores prior to their extinction soon after initial human settlement. Here we contribute to the knowledge of moa diet and ecology by reporting the results of a multidisciplinary study of 35 coprolites from a subalpine cave (Euphrates Cave) on the South Island of New Zealand. Ancient DNA analysis and radiocarbon dating revealed the coprolites were deposited by the extinct upland moa (*Megalapteryx didinus*), and span from at least 6,368±31 until 694±30 ^14^C years BP; the approximate time of their extinction. Using pollen, plant macrofossil, and ancient DNA analyses, we identified at least 67 plant taxa from the coprolites, including the first evidence that moa fed on the nectar-rich flowers of New Zealand flax (*Phormium*) and tree fuchsia (*Fuchsia excorticata*). The plant assemblage from the coprolites reflects a highly-generalist feeding ecology for upland moa, including browsing and grazing across the full range of locally available habitats (spanning southern beech (*Nothofagus*) forest to tussock (*Chionochloa*) grassland). Intact seeds in the coprolites indicate that upland moa may have been important dispersal agents for several plant taxa. Plant taxa with putative anti-browse adaptations were also identified in the coprolites. Clusters of coprolites (based on pollen assemblages, moa haplotypes, and radiocarbon dates), probably reflect specimens deposited at the same time by individual birds, and reveal the necessity of suitably large sample sizes in coprolite studies to overcome potential biases in diet interpretation.

## Introduction

Large herbivores are key components of terrestrial ecosystems [1], providing essential ecosystem services such as seed dispersal, pollination and nutrient cycling. They also play a major role in shaping vegetation community composition and structure, and influencing fire regimes [2]–[5]. The widespread extinction of many large herbivores during the late Pleistocene and Holocene has resulted in the loss of the ecosystem processes provided by these animals, fundamentally altering the functioning of ecosystems across the globe [5]–[8]. Understanding the wider ecological implications of these extinctions relies partly on detailed information about the habitats and diets of extinct herbivores. Such information can be obtained in several ways, including analysis of skeletal morphology [9], tooth-wear [10], bone isotopes [11] and coprolites [12].

Perhaps the most comprehensively studied of all extinct megafaunal herbivores are the New Zealand moa (Aves: Dinornithiformes) [13]. Nine species of moa in six genera [14], [15], some weighing more than 250 kg [16], were New Zealand’s largest native herbivores [17] but became extinct shortly after initial human settlement in the 13th Century AD [18]. Direct evidence for diet currently exists for all of the six moa genera, in the form of preserved gizzard content associated with skeletons in miring bone deposits (*Dinornis, Emeus, Euryapteryx,* and *Pachyornis*) [19]–[22] and coprolites from caves and rock shelters (*Dinornis, Pachyornis, Euryapteryx, Megalapteryx,* and *Anomalopteryx*) [23]–[25]. Despite the relative plethora of well-preserved fossil remains, there are still many unanswered questions surrounding the paleoecology of moa, particularly with respect to diet, habitat use, and niche partitioning.

**Figure 1:**
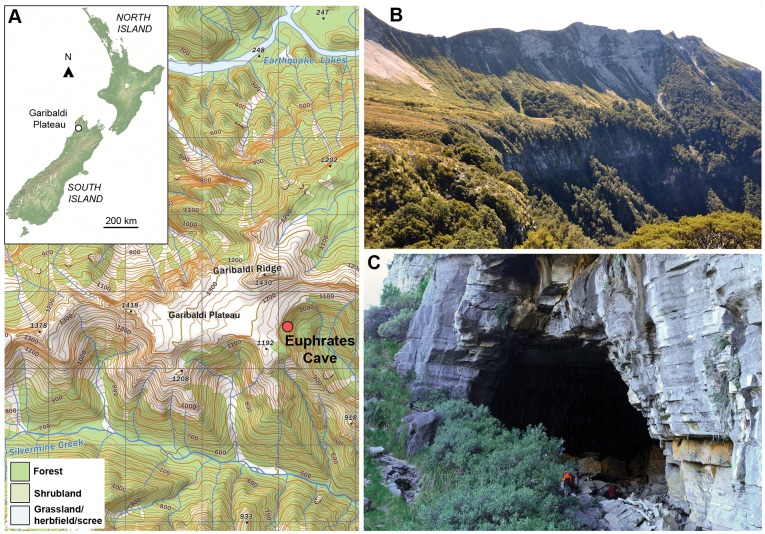
Location and environment of Euphrates Cave. (A) Main vegetation types near Euphrates Cave, South Island, New Zealand. Grids are 1 km^2^. (B) View north from eastern end of Garibaldi Plateau, showing Garibaldi Ridge, and the eastern buttress below the plateau. (C) Main entrance of Euphrates Cave at the base of the eastern buttress (note people for scale).

In recent years, issues such as the possible effects of moa extinction on vegetation dynamics, and the role of moa herbivory in the evolution of potential anti-browsing traits in the New Zealand flora, have generated considerable debate in the literature [17], [24], [26]–[34]. Moreover, the relatively recent extinction of moa means that the ecological impact of their loss is yet to be fully realised. Some long-lived trees that may have had their juvenile branches browsed upon by moa, or grown from seeds dispersed in moa dung, may still be present in forests today. Additional paleoecological data on moa diets are therefore critical for a deeper understanding of how their extinction has impacted New Zealand’s native vegetation communities.

Recently, several accumulations of moa coprolites have been excavated and analysed from mostly lowland rock shelters on the South Island of New Zealand [24], [25], [35], providing new data on the diet and ecology of these large herbivorous birds. Here, we examine a new sample of moa coprolites from a subalpine cave in the northwest corner of the South Island; an environment and region from which the diets of moa remain virtually unknown. We use a multiproxy approach, combining analyses of macrofossils (seeds and leaf fragments), microfossils (pollen), inorganic content, ancient DNA, and radiocarbon dating, to provide new insights into the diet and ecology of moa in the subalpine zone.

## Methods

### Study Site (Euphrates Cave)

The largest entrance to Euphrates Cave [36] is located at the treeline (c. 1000 m elevation), at the base of a buttress cliff demarcating the eastern end of the Garibaldi Plateau (41° 14′ 17′′ S, 172° 25′ 49′′ E), in Kahurangi National Park, South Island, New Zealand (Figs. 1a, 1b). Due to its relative remoteness and inaccessibility, the cave has rarely been visited since it was discovered in 1994 [36]. The main passage of the cave extends approximately 1.5 km southwest of the large entrance (Fig. 1c) [36], and is formed within Oligocene calcareous mudstones and sandstones of the Matiri Formation [37].

In February 2010 we discovered an accumulation of moa coprolites immediately within the large entrance to the cave. Several of the coprolites were found on the surface of rocks, although most of those collected (approximately 100) were in unconsolidated dry sand between blocks of roof-collapse debris. The site received sunlight and was subject to a constant breeze; both factors probably contributed to the desiccation and preservation of the coprolites.

Tall forest occurs within 30 m of the main cave entrance, and extends down to the valley floor below and in small areas above the buttress (Fig. 1). The dominant canopy species is silver beech (*Nothofagus menziesii*), with subcanopy shrubs including neinei (*Dracophyllum traversii*), weeping matipo (*Myrsine divaricata*), mountain toatoa (*Phyllocladus alpinus*), *Pseudopanax linearis,* and *Coprosma* spp. [38]. Around the entrance to the cave, and along the base of the buttress, there is a narrow margin of open vegetation dominated by grasses (*Chionochloa* and *Poa* spp.), tree daisy (*Olearia colensoi*), *Veronica,* and mountain flax (*Phormium cookianum*). The vegetation on the plateau above the buttress is largely subalpine grassland (red tussock, *Chionochloa rubra*), with diverse herbs and small shrubs, particularly around steep rock outcrops and on the inside walls of sinkholes that are likely to be inaccessible to introduced herbivores such as red deer (*Cervus elaphus*), European hare (*Lepus europaeus*) and brushtail possum (*Trichosurus vulpecula*). A complete list of plant species from the Garibaldi Range is given by Druce et al. [38].

### Coprolite Sampling

Thirty-five intact coprolites (Fig. S1) were selected for this study from the c. 100 collected from Euphrates Cave. Subsampling of the coprolites was performed in a still-air perspex hood. To prevent cross-contamination, surfaces within the hood were cleaned between samples with 10% decon, 2% bleach, and ethanol. To control for post-deposition environmental contamination, approximately 2–3 mm from the surface of each coprolite was removed by scraping with a scalpel blade, and the freshly exposed surface was UV irradiated for 15 minutes. The specimens were then broken in half, and subsamples for pollen and DNA analysis were taken from the interior of each coprolite. Additional samples were taken for inorganic content analysis, radiocarbon dating, and voucher specimens (held at Landcare Research, Lincoln, New Zealand). The remainder of each coprolite was analysed for macrofossils.

### Ancient DNA Analyses

#### DNA extraction, moa DNA amplification and sequencing

DNA extraction and PCR setup was performed in a geographically isolated ancient DNA laboratory at the Australian Centre for Ancient DNA (ACAD), University of Adelaide. PCR amplification and all downstream procedures were carried out in the ACAD post-PCR laboratory. Strict ancient DNA procedures were followed to minimise contamination of samples with exogenous DNA [39], including the use of multiple negative extraction and amplification controls to detect contamination.

Subsamples from the interior of each coprolite were rehydrated for 24 hours in 10 mM Tris-HCl, (pH 8.0), and DNA was extracted from 0.25–0.30 g of rehydrated coprolite using the MoBio Power Soil Kit following the manufacturer’s protocol.

To determine the identity of the depositing moa species, a 31 base-pair (bp) fragment (excluding primers) of the mitochondrial DNA control region was amplified using the moa-specific primers 262F and 294R [40]. 262F included a M13USP-tag on the 5′ end (5′ TGTAAAACGACGGCCAGT 3′) to increase the total amplicon length and improve sequencing reads on short DNA fragments (following [24]). The 31 bp fragment discriminates all nine recognised moa species with the exceptions of little bush moa (*Anomalopteryx didiformis*) and coastal moa (*Euryapteryx curtus*) [24], [41].

PCR reactions (total volume 25 µL) contained 2 mg/mL RSA (Sigma), 1 × PCR buffer (Platinum, Invitrogen), 2 mM MgSO_4_, 2.5 µM each dNTP, 0.4 µM each primer, 1 unit Platinum Taq HiFi (Invitrogen), and 2 µL DNA. The PCR cycling conditions were: 94°C 3 min; 55 cycles of 94°C 30 s, 55°C 30 s and 68°C 45 s; 68°C 10 min.

PCR products were visualised on a 3.5% 1 × TBE agarose gel. The PCR products were then purified using EXOSAP (4 units Exo1, 0.6 units SAP; incubation at 37°C for 30 min and 80°C for 15 min) and sequenced with the M13USP primer using Big Dye Terminator technology (BigDye v3.1) and separated on an ABI 3130XC capillary sequencer.

#### Plant DNA amplification, cloning and sequencing

To identify the dietary plant species in a subsample of 12 coprolites, a 95 bp fragment (excluding primers) of the chloroplast encoded gene *rbcL* was amplified using the universal angiosperm primers h1aF and h2aR [42], and the PCR reagent and cycling conditions described above. PCR products were visualised on a 3.5% 1 × TBE agarose gel, and purified using the AMPure magnetic bead system (Agencourt) following the manufacturer’s instructions. PCR products were cloned using the Strataclone PCR cloning kit (Agilent, Stratagene). Blue-white screening of colonies was performed using X-gal. White colonies containing the *rbcL* insert were picked and added directly to PCR tubes containing a 25 µL volume of 1 × PCR buffer (Hotmaster, Eppendorf), 2 µM each dNTP, 0.4 µM each primer (M13–20, 5′ GTAAAACGACGGCCAG 3′ and M13RSP, 5′ CAGGAAACAGCTATGACCAT 3′), and 1 unit of Hotmaster Taq (Eppendorf). PCR cycling conditions were 94°C 12 min; 35 cycles of 94°C 20 s, 55°C 10 s and 65°C 45 s; 65°C 10 min. PCR products were visualised on a 2% 1 × TBE agarose gel and purified using EXOSAP as described above. Twenty four to 48 clones were sequenced using the M13–20 primer and Big Dye Terminator technology (BigDye v3.1), and separated on an ABI 3130XC capillary sequencer. Although pollen of some angiosperms contain plastids, in the majority of angiosperms (>80% of species [43]) they are inherited along the maternal line. Therefore the DNA obtained from the coprolites using the primers h1aF and h2aR will mostly represent ingested plant tissues such as leaves, twigs, fruit rather than pollen.

#### DNA sequence analyses

Moa DNA sequences were identified to species through comparison with a reference database comprising 164 control region sequences from vouchered moa specimens of known taxonomic identity (obtained from Genbank) (following [24], [41]). These were analysed using the BEAST package v. 1.6.1 [44] following the method described below for analysis of the *rbcL* sequences.

Eighty two unique *rbcL* clone sequences (95 bp) were obtained from the moa coprolites. These were tentatively assigned to taxa using BLAST similarity matches of >96%. To more robustly identify dietary plant taxa, the *rbcL* sequences were aligned, using the Clustal W algorithm in MEGA 4.0 [45], with 160 reference *rbcL* sequences (sourced from unpublished data sets and Genbank, including tentative BLAST identifications) comprising the full *rbcL* gene (1402 bp) and representing the full range of plant taxa presently found in the mountains near Euphrates Cave [38].

The *rbcL* alignment was analysed using BEAST 1.6.1 [44]. A Yule speciation prior and a relaxed lognormal clock on the nucleotide substitution rate were used, as recommended for multi-species phylogenetic analysis [44]. The nucleotide substitution model (HKY + G (four categories)) was determined using ModelTest and the Aikaike Information Criterion. To account for possible post-mortem DNA damage an age-independent transitions only model of sequence evolution was implemented [46]. The analysis was run for 25 million generations, logging parameters every 1000 generations. Convergence was assessed using Tracer v1.5 [47]. LogCombiner and TreeAnnotator [44] were used to combine and summarize the information in the tree output file, and a maximum clade credibility tree with posterior probability values was drawn using FigTree [48].

### Pollen

Subsamples (0.08–0.54 g; mean = 0.39 g) from the interior of each coprolite were prepared for pollen analysis. First the samples were disaggregated by soaking in hot KOH for 10 minutes, then passed through a 100 µm sieve (to remove coarse organics) and treated with cold10% HCl to remove calcium carbonate. This was followed by acetolysis (samples were treated with acetic anhydride and sulphuric acid) for 3 minutes to remove cellulose. Finally, inorganic particles were removed using lithium polytungstate flotation (at specific density 2.2), after which the samples were stained with fuchsin and mounted in glycerine jelly on a microscope slide. Prior to the acetolysis step, each sample was spiked with a known number of exotic *Lycopodium* spores to allow determination of pollen concentrations. Counts of at least 250 pollen/spores were made for each sample. We identified pollen taxa using the standard New Zealand nomenclature [49]. Cluster analysis of coprolite pollen assemblages was performed using the program Gene Cluster [50]. Within the natural environment, pollen types of different plants are present in varying amounts due to their pollination mode, the amount of pollen produced, and dispersability of pollen grains [49]. This has the potential to bias pollen assemblages in coprolites, so that they do not necessarily reflect relative abundance of dietary components (for example, wind dispersed pollen present on the surface of consumed leaves). To overcome this potential bias we assigned an environmental prevalence index (EPI) to each identified pollen type and analysed ranked pollen abundance with respect to EPI values. The EPI was calculated for each pollen taxon by multiplying values for pollination mode (wind = 3; insect or other animals = 1), pollen production, and dispersability (values given by [49]). EPI values range from 1 (animal pollinated, low pollen production, restricted dispersal) to 27 (wind pollinated, prolific pollen production, wide dispersal). Thus, pollen types that were overrepresented in the coprolites, relative to their expected environmental prevalence (plotting above the null, or cumulative frequency, distribution), were more likely to reflect dietary components. Those that were underrepresented (plotting below the null distribution) were more likely to reflect incidental, or rare, ingestion.

### Plant Macrofossils

Portions of coprolites for macrofossil analysis were rehydrated for at least two weeks in 0.5% trisodium phosphate buffer, and sieved on 0.25 mm mesh. The retained residue was examined under a microscope at 8–20× magnification. Seeds, leaf fragments and invertebrate remains were picked out of the residue using fine forceps and stored in vials containing 70% ethanol. Identifications were made based on photographs and descriptions [51] and by comparison with modern reference material in the Allan Herbarium (CHR), Landcare Research.

### Organic Content

Organic content was measured using the loss on ignition method [52]. Coprolite subsamples (0.071–1.05 g) were dried for 24 hours at 90°C, weighed, and ashed in a muffle furnace for 4 hours at 550°C. Organic content was calculated by determining the weight loss after combustion as a percentage of the original dry weight of each sample.

### Radiocarbon Dating

Subsamples from the insides of 12 coprolites were submitted to the Waikato Radiocarbon Laboratory, New Zealand, for AMS dating. The samples were washed in hot HCl, then rinsed and treated with multiple hot NaOH washes. The NaOH insoluble fraction was treated with hot HCl, filtered, rinsed and dried. Radiocarbon ages were calibrated using the SHCal04 curve [53] in OxCal v.4.1 [54] and are reported as the 95.4% confidence range.

## Results

### Moa Species

The 31 bp fragment of moa mtDNA control region was successfully amplified and sequenced from 33 (94.3%) of the 35 coprolites. All sequences were attributable to upland moa (*Megalapteryx didinus*) (Fig. S3). The identification of upland moa as the depositing species is congruent with both the inferred subalpine habitat of the species [16], and the presence of upland moa bones in caves on Garibaldi Plateau. Two haplotypes were identified within the 33 sequences, differing by a C (n = 12) or T (n = 21) at nucleotide position 25 (Fig. S2). Although C–T transitions caused by the deamination of cytosine are a common artefact of post-mortem ancient DNA damage [55] (Hofreiter et al. 2001), we believe that in this case they reflect genuine haplotype variationbecause: 1) both the C and T haplotypes are geographically consistent, having previously been sequenced from upland moa remains from the north-west Nelson/West Coast regions (Figs. S2; Figs. S3; [14]); and 2) identical haplotypes were obtained from different coprolites that were probably deposited by individual birds, based on shared radiocarbon ages and pollen assemblages (discussed below).

### Plant DNA

Ancient plant DNA was obtained from 67% (8 of 12 sampled) coprolites, with between 16 and 52 *rbcL* clones sequenced per sample (199 in total). The aligned dataset is given in Fig. S4. Anomalous changes to the highly-conserved amino acid sequence of *rbcL* (including an internal stop-codon), suggested a small amount of post mortem DNA damage was evident in the sequences. Overall, there was strong agreement between clone identifications using the BLAST and the BEAST (Fig. S5) analyses, though some clones with 100% BLAST matches (e.g. *Nothofagus menziesii*) had low posterior probability support in the BEAST analysis. This is probably due to multiple unique sequences from the same plant taxon varying by only 1–2 bp, obscuring their exact placement within the tree. Low posterior probabilities were also obtained where taxonomically distinct plant taxa shared similar sequences for the 95 bp *rbcL* fragment (e.g. *Griselinia, Schefflera* and *Quintinia*). Nonetheless the BEAST analysis of the *rbcL* dataset was able to identify sequences that did not return close matches using BLAST. A summary of the 27 plant taxa identified from DNA is given in Table 1.

**Table 1:** Plant taxa identified in upland moa coprolites from Euphrates Cave.

	Family	Common name	Scientific name	DNA	Pollen	Macrofossils
**Trees and** **shrubs**	Podocarpaceae	Rimu	*Dacrydium cupressinum*	N/A	29/35 (0–**0.5**–2.5)	
		Miro	*Prumnopitys ferruginea*	N/A	18/35 (0–**0.3**–1.5)	
		Matai	*P. taxifolia*	N/A	26/35 (0–**0.6**–2.6)	
		Totara	*Podocarpus*	N/A	17/35 (0–**0.2**–1.2)	
			*Halocarpus*	N/A	2/35 (0–**0.02**–0.3)	
		Toatoa	*Phyllocladus*	N/A	15/35 (0–**0.3**–1.8)	
	Fagaceae	Silver beech	*Nothofagus menziesii*	(5/8)	30/35 (0–**1.3**–3.6)	*L* (6/35)
			Fuscospora (pollen type)		35/35 (0.3–**4.8**–15.2)	
	Myrtaceae	Rata	*Metrosideros*		4/35 (0–**0.04**–0.4)	
		Rōhutu	*Neomyrtus*		6/35 (0–**0.1**–0.9)	
	Araliaceae		*Pseudopanax*	(1/8)**	4/35 (0–**0.1**–1.4)	
		Mountain five-finger	*P. colensoi*		8/35 (0–**0.6**–16.9)	
	Onagraceae	Tree fuchsia	*Fuchsia excorticata*	(1/8)	9/35 (0–**0.3**–3.8)	*S* (2/35)
	Chloranthaceae	Hutu	*Ascarina lucida*		2/35 (0–**0.01**–0.3)	
	Paracryphiaceae	Quintinia	*Quintinia*	(1/8)		
	Elaeocarpaceae	Wineberry	*Aristotelia*		1/35 (0–**0.01**–0.3)	
			*Elaeocarpus/Aristotelia*	(7/8)		
	Grisiliniaceae	Broadleaf	*Griselinia*	(4/8)	1/35 (0–**0.01**–0.4)	
	Cunoniaceae	Tōwai	*Weinmannia*	(2/8)		
	Piperaceae	Kawakawa	*Macropiper*	(1/8)		
	Myrsinaceae	Māpou	*Myrsine*	(2/8)	5/35 (0–**0.1**–0.9)	
	Rubiaceae	Karamū	*Coprosma*		18/35 (0–**0.9**–8.6)	
		Mamangi	*Coprosma* cf. *arborea*	(1/8)**		
			Undetermined	(3/8)**		
	Plantaginaceae	Veronica	*Parahebe*		1/35 (0–**0.01**–0.4)	
		Veronica	*Veronica*	(1/8)**		
	Ericaceae		Undetermined	(1/8)	10/35 (0–**0.4**–6.8)	
		Snowberry	*Gaultheria*	(1/8)**		*S* (10/35)
		Grass tree	*Dracophyllum*	(1/8)		
	Epacridaceae		Undetermined			*L* (1/35)
	Coriariaceae	Tutu	*Coriaria*	(1/8)**		
**Lianes**	Rosaceae	Bush lawyer	*Rubus*		1/35 (0–**0.01**–0.3)	
	Polygonaceae	Pōhuehue	*Muehlenbeckia*		1/35 (0–**0.02**–0.7)	
**Dicotyledonous**	Asteraceae	Daisy	Undetermined	(1/8)**	33/35 (0–**5.9**–32.5)	
**herbs**			Lactuceae		18/35 (0–**0.9**–6.5)	
	Rosaceae	Bidibidi	*Acaena*	(2/8)**	19/35 (0–**1.5**–17.3)	
	Thymeleaceae	Rice flower	*Pimelea*		1/35 (0–**0.01**–0.3)	
			*Kelleria*		1/35 (0–**0.01**–0.3)	
	Brassicaceae		Undetermined	(1/8)**	26/35 (0–**1.5**–7.5)	
	Lobeliaceae		cf. *Pratia*			*S* (4/35)
	Onagraceae	Willowherb	*Epilobium*		14/35 (0–**0.1**–1.0)	
	Gentianaceae	Gentian	*Gentiana*		25/35 (0–**3.2**–14.6)	
			Undetermined	(1/8)*		
	Droseraceae	Sundew	*Drosera* spp.		5/35 (0–**0.1**–1.4)	
	Plantaginaceae	Plantain	*Plantago*		18/35 (0–**0.6**–2.8)	
	Caryophyllaceae	Colobanthus	*Colobanthus*			*S* (2/35)
			Undetermined		16/35 (0–**0.1**–0.4)	
	Oxalidaceae	Oxalis	*Oxalis*	(2/8)		
	Boraginaceae	Forget-me-not	*Myosotis*	(2/8)*	32/35 (0–**1.6**–7.7)	
	Halagoraceae	Milfoil	*Myriophyllum*		1/35 (0–**0.01**–0.3)	
	Donatiaceae		*Donatia*		2/35 (0–**0.02**–0.3)	
	cf. Urticaceae	Nettle	cf. *Urtica*			*S* (5/35)
	Ranunculaceae	Buttercup	*Ranunculus*	(1/8)**	24/35 (0–**0.9**–4.9)	*S* (10/35)
	Stylidiaceae		*Forstera/Phyllachne*	(1/8)		
	Loganiaceae		*Mitrasacme*	(1/8)		
	Apiaceae		Undetermined		20/35 (0–**4.2**–27.4)	
		New Zealand carrot	*Anisotome*		3/35 (0–**0.2**–6.1)	
	Violaceae	Violet	*Viola*	(1/8)**		
**Monocotyledons**	Poaceae	Grass	Undetermined	(1/8)**	35/35 (0.7–**36.4**–86.0)	*S* (1/35), *Fl* (3/35)
	Cyperaceae	Sedge	Undetermined	(2/8)**	27/35 (0–**5.7**–64.7)	
			cf. *Carex*			*S* (3/35)
			cf. *Scirpus*			*S* (13/35)
	Asteliaceae	Mauri	*Astelia*		20/35 (0–**7.2**–84.1)	
	Xanthorrhoeaceae	Bulbine lily	*Bulbinella*		9/35 (0–**0.1**–1.7)	
		New Zealand flax	*Phormium*		14/35 (0–**2.9**–76.7)	
**Ferns and**			Unidentified fern	N/A		*L* (3/35)
**allies**			Monolete fern	N/A	34/35 (0–**7.3**–32.3)	
	Cyatheaceae	Mountain tree fern	*Cyathea colensoi*	N/A	26/35 (0–**4.0**–38.6)	
	Hymenophyllaceae	Filmy fern	*Hymenophyllum*	N/A	9/35 (0–**0.3**–3.9)	
	Ophioglossaceae	Adder’s tongue	*Ophioglossum*	N/A	15/35 (0–**0.5**–3.8)	
	Lycopodiaceae	Club moss	*Lycopodium australianum*	N/A	2/35 (0–**0.1**–1.8)	
		Creeping club moss	*L. scariosum*	N/A	2/35 (0–**0.1**–2.9)	
	Anthocerotaceae	Hornwort	*Anthoceros*	N/A	1/35 (0–**0.01**–0.3)	
**Bryophyta**	Undetermined	Moss		N/A		*L* (17/35)

N/A: non-angiosperm taxa unable to be detected by DNA primers used in this study. DNA taxa are nearest matches in Bayesian analysis, and presented as number of coprolites in which the taxa occurred. ** >90% posterior probability (p.p.) support; * 80–90% p.p. support. Pollen data are number of coprolites in which the taxa occurred and (min. - mean - max.) percentages of total pollen sum per coprolite. Macrofossil (*Fl,* florets; *L*, leaf; *S*, seeds) data are number of coprolites in which the taxa occurred.

**Figure 2:**
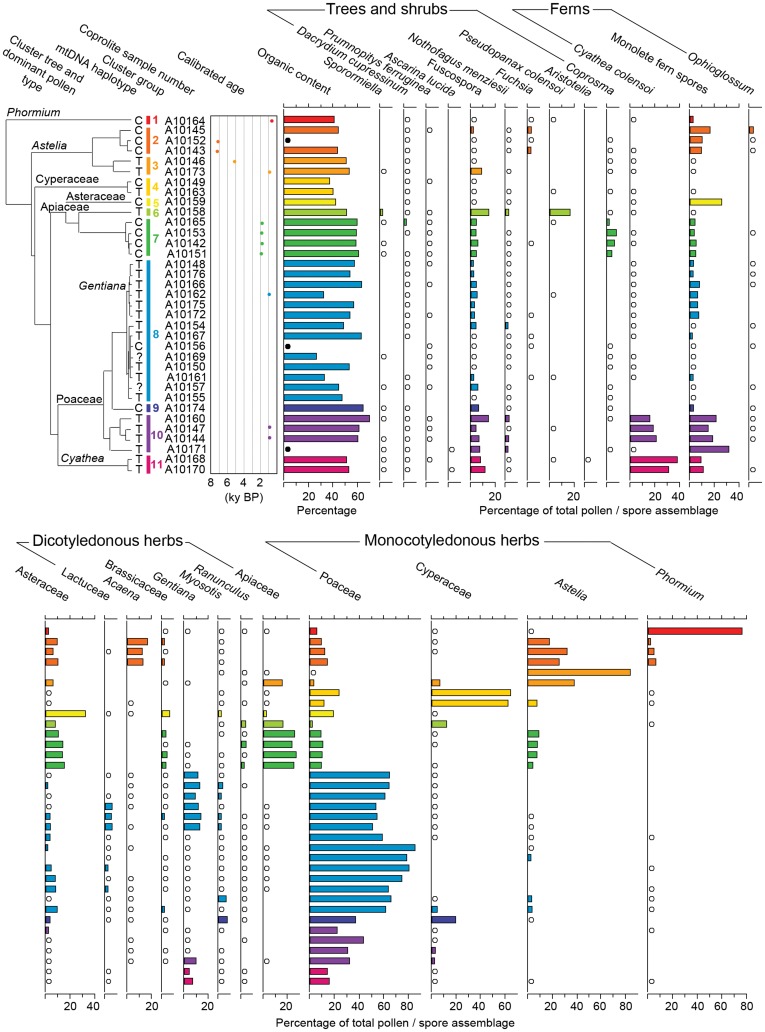
Pollen diagram for upland moa coprolites from Euphrates Cave. Pollen assemblages are plotted with cluster analysis tree of coprolites based on pollen assemblages, calibrated ages of radiocarbon dated coprolites, and organic content. Colouring defines coprolites sharing highly similar pollen assemblages. Open circles represent presence at trace amounts (<2.5%). Black circles represent samples where there was insufficient sample to analyse organic content.

**Figure 3:**
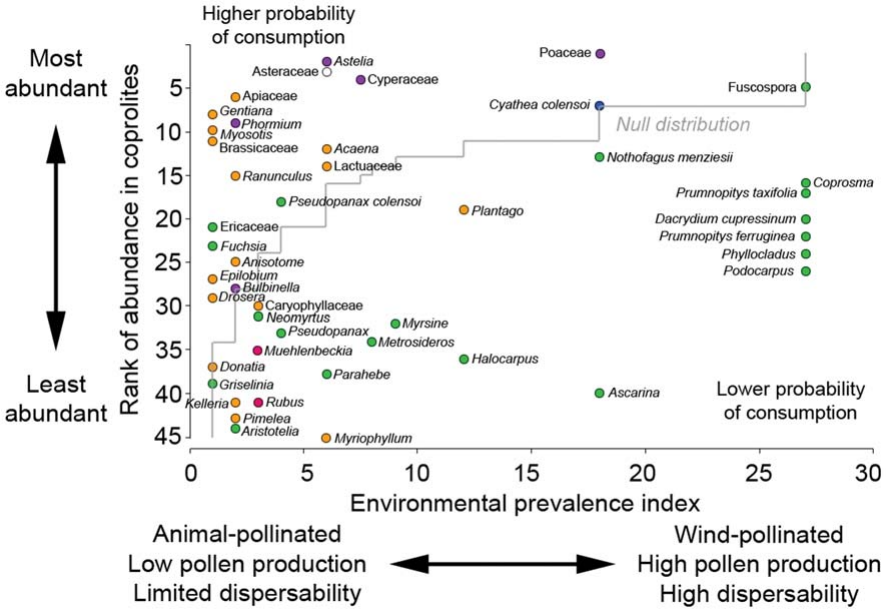
Ranked abundance of pollen/spore types in upland moa coprolites from Euphrates Cave vs. environmental prevalence index. Ranked abundances are based on the summed percentages of each pollen taxon from all 35 coprolites (1 =  most abundant pollen/spore type, 45 =  least abundant pollen/spore type). Ground ferns, *Lycopodium* spp., and *Anthoceros* are not included as data used to calculate environmental prevalence index values were not provided for these taxa by [49]. The null (cumulative frequency) distribution line represents the hypothetical distribution of the pollen taxa if their abundance in the coprolites was directly related to their environmental prevalence. Pollen taxa plotting above this line are overrepresented in coprolites relative to their environmental prevalence, suggesting they may have been directly eaten. Pollen taxa plotting below this line are underrepresented in the coprolites, suggesting they may have been eaten more rarely, or incidentally ingested. Circle colours reflect plant types: green, trees and shrubs; orange, dicot herbs; pink, lianes; purple, monocot herbs; blue, ferns; white, undeterminable.

### Pollen

**Figure 4:**
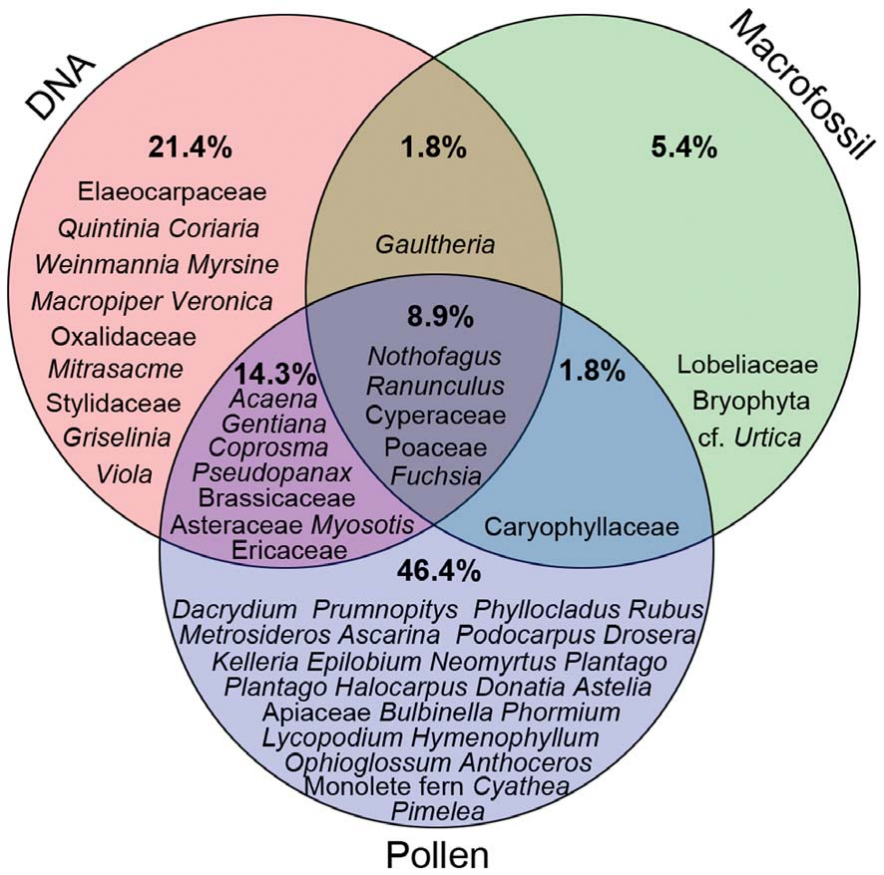
Plant taxa detected in upland moa coprolites from Euphrates Cave using different proxies. Plant taxa detected in eight upland moa coprolites from Euphrates Cave for which all 3 proxies (plant ancient DNA, macrofossils, pollen) were used.

Pollen and spores from 51 plant taxa were identified in the coprolites (Tables 1; S1). In addition spores of the dung-fungi *Sporormiella* were recorded in 13 (37%) of the coprolites (Fig. 3). The majority of plant taxa represented by pollen/spores in the coprolites are typical of either *Nothofagus* forest or subalpine grassland, the dominant habitat types near Euphrates Cave today. Cluster analysis identified several groups of coprolites sharing highly similar pollen assemblages (Fig. 2). The most frequently occurring pollen/spore types in the coprolites were Fuscospora (a subgenus of *Nothofagus* that includes *N. fusca*, *N. solandri* s.l. and *N.* *truncata*) and grasses (Poaceae) (observed in 100% of coprolites), followed by monolete ferns (97.1%), daisies (Asteraceae) (94.3%), forget-me-not (*Myosotis*) (91.4%) and *Nothofagus menziesii* (85.7%). Dominant pollen/spore types within individual coprolites were New Zealand flax (*Phormium*), mauri (*Astelia*), sedges (Cyperaceae), Asteraceae, Apiaceae, Poaceae, and mountain tree fern (*Cyathea colensoi*) (Fig. 2). Distinguishing pollen types that represent dietary items from those that are environmental is difficult, although plotting the ranked abundance of pollen types against an index of environmental prevalence (Fig. 3) provides a crude guide. Pollen types that are overrepresented in the coprolites with respect to their environmental prevalence are likely to reflect plant taxa eaten by moa. This is supported by the observation that plant taxa identified by all three proxies (DNA, pollen, and macrofossil) (Fig. 4), and thus undoubtedly consumed by moa, were all overrepresented in the coprolite pollen assemblages; plotting above the null distribution line (Fig. 3). The most overrepresented include *Astelia*, Asteraceae (including Lactuceae), Cyperaceae, Apiaceae, gentian (*Gentiana*), *Phormium*, *Myosotis*, Brassicaceae, bidibid (*Acaena*), buttercup (*Ranunculus*) and mountain five-finger (*Pseudopanax colensoi*) (Fig. 3). Pollen types that are underrepresented in the coprolite pollen assemblages include wind-dispersed pollen types from plant taxa that are locally rare, or do not occur near Euphrates Cave e.g. hutu (*Ascarina*), miro (*Prumnopitys ferruginea*), and *Dacrydium* (Fig. 3). Although this approach is somewhat subjective (as it is based on assigning values to pollination characteristics) it provides a new method for assessing dietary vs. environmental components that seems to be supported by independent proxies. Actual measurements of local environmental pollen prevalences (using surface sampling) may improve the method, but would vary seasonally.

### Plant Macrofossils

The coprolites were comprised mostly of fine amorphous material probably representing well-digested plant tissues (as noted by [24]). However, seeds and leaf fragments from at least 13 plant taxa were identified in the coprolites (Tables 1; S2), including leaves of *Nothofagus menziesii*, Epacridaceae, fern and moss, and seeds of *Fuchsia*, snowberry (*Gaultheria*), Lobeliaceae (cf. *Pratia*), *Colobanthus*, cf. nettle (*Urtica*), *Ranunculus*, Poaceae, and at least 2 different Cyperaceae (cf. *Carex* and cf. *Scirpus*). The seed assemblage is similar to that reported from coprolites of four moa species by Wood et al. [24]. Seeds were present in 77.1% of the coprolites, and most of the identified seeds were intact, suggesting they could potentially have been dispersed by upland moa. As with Wood et al. [24], we also noted occasional small invertebrate fragments (Coleoptera, Diptera, Acari) that probably originated either post-deposition, or through incidental ingestion with plant matter.

### Organic Content

The organic content of the coprolites varied significantly, ranging from 27.2–69.8% (mean = 51.4%; standard error = 1.8%). These values are consistent with those (28–60%) reported for coprolites of other large herbivores, e.g. Svalbard reindeer (*Rangifer* spp.) [56]. The inorganic component in the moa coprolites probably reflects a combination of mineral-rich soil ingested during grazing, biogenic silica from browsed plant matter, and particulate material derived from ground gizzard stones. Quartz grains, commonly fine sand and silt size, but up to 5.5 mm diameter, were observed within the coprolites.

### Radiocarbon Dating

Radiocarbon dates for the coprolites (Table 2) ranged from 6368±31 yrs BP (7315–7165 cal. BP) to 694±30 yrs BP (663–559 cal. BP). The earliest dates represent the oldest known radiocarbon dated moa coprolites; all previously studied specimens have been late Holocene in age [23]–[25]. The youngest dates for the Euphrates Cave moa coprolites are within the post-settlement era and around the time of moa extinction [18]. Three distinct clusters of multiple coprolites with similar ages were evident (Fig. 2): A10143 and A10152 (6368 and 6310 yrs BP); A10142, A10151, A10153 and A10165 (1964–1879 yrs BP); and A10144, A10147, A10162 and A10173 (1060–975 yrs BP).

**Table 2:** Radiocarbon dates for upland moa coprolites from Euphrates Cave.

Sample no.	RadiocarbonLab no.	Conventional radiocarbon age(CRA) (yrs BP)	CRA error	δ^13^C	*Calibrated age yrs BP(95.4% confidence)
A10164	Wk28344	694	30	−29.9±0.2	663−559
A10173	Wk28346	975	30	−29.1±0.2	920−772
A10144	Wk29439	997	26	−28.6±0.2	923−798
A10147	Wk28341	1000	30	−29.0±0.2	927−796
A10162	Wk28343	1060	30	−31.2±0.2	967−811
A10165	Wk28345	1879	30	−29.4±0.2	1865−1632
A10142	Wk28340	1915	30	−29.4±0.2	1875−1713
A10153	Wk29442	1959	25	−29.1±0.2	1927−1735
A10151	Wk29441	1964	25	−29.3±0.2	1929−1739
A10146	Wk29440	4520	28	−29.9±0.2	5290−4968
A10152	Wk28342	6310	31	−30.2±0.2	7266−7022
A10143	Wk29438	6368	31	−30.3±0.2	7315−7165

All radiocarbon dates are based on samples of bulk material taken from inside of coprolites. *Calibrations based on ShCal04 curve [53].

**Figure 5:**
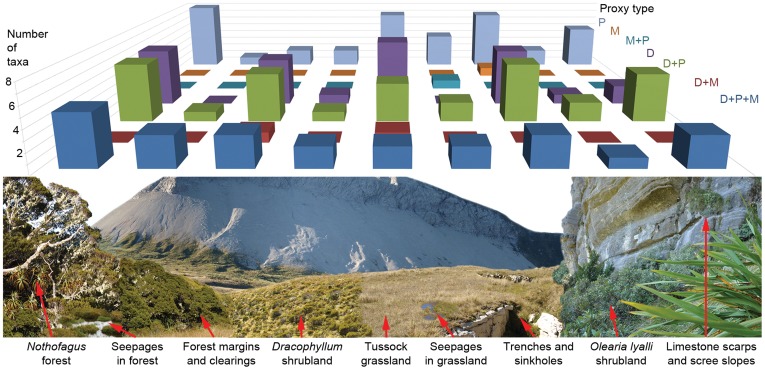
Vegetation communities represented by plant taxa in upland moa coprolites from Euphrates Cave. Occurrence of plant taxa identified in upland moa coprolites from Euphrates Cave within the nine major vegetation communities presently occurring near the cave (based on [38] and authors observations). Single plant taxa can occur in multiple vegetation communities. Identified plant taxa are also grouped according to proxies with which they were detected (P, pollen; M, macrofossil, D, ancient DNA).

## Discussion

### Authenticity of the Coprolite DNA

Controlling for potential contamination is an important aspect of ancient DNA research. Post-excavation contamination can be controlled by taking appropriate precautions such as storing samples in sterile packaging and working on specimens in dedicated ancient DNA facilities. However, pre-excavation contamination from environmental DNA, such as that in the surrounding soil or sediment, is also a concern. The issue of DNA leaching causing contamination of coprolites has previously been raised [57], however experiments have led to the conclusion that “*substantial amounts of liquid water are required to move free DNA molecules between strata*” [58]. While DNA leaching may therefore be an issue for some soils and damp cave sediments, it is unlikely to affect desiccated coprolites due to the fact they rely on arid conditions for preservation. Further, several additional lines of evidence suggest the DNA from the Euphrates Cave coprolites is authentic and not affected by in-leaching. First, the sediment in which the coprolites were deposited was an inorganic white limestone powder comprised of weathering products derived from the roof and walls of the cave. There was no soil or organic-derived sediment (e.g. leaf litter) at the site. Second, the coprolites were located sufficiently distant (>20 m) from the zone of plant growth that there was no contamination risk from fine rootlets penetrating the samples. Third, the coprolites were so dry they had attained hydrophobic properties, and therefore would have been difficult for water to penetrate had there been any moisture present at the site. Finally, the coprolites contained a diverse range of plant DNA (with assemblages varying between samples), from both forest and herbfield taxa. Many of the plants represented by DNA were present as pollen or macrofossils in the samples, or have been previously been identified in moa diets.

### Accumulation of the Coprolite Deposit

Through examining the clustering of coprolites based on pollen assemblages, mtDNA haplotypes, and radiocarbon dates (Fig. 2), we can make some inferences about the rate of accumulation and the minimum number of individual birds represented in our sample. Three coprolite clusters (groups 2, 7, and 10 in Fig. 2) share nearly identical pollen assemblages and are of a single moa haplotype. Each of these clusters probably represent coprolites that were deposited by one bird in a single defecation event. This is supported by individual coprolites from within these clusters sharing overlapping radiocarbon age distributions. At least four of the sampled coprolites were comprised of multiple boli that were presumably excreted together and remained attached post-desiccation (A10165, A10157, A10172, and A10162 in Fig. S1). Coprolites A10174 and A10162 share the same radiocarbon age and mtDNA haplotype as group 10, but have different pollen assemblages (Fig. 2), and therefore may represent coprolites deposited by the same bird on different days. Coprolite A10162 is itself within a cluster of 3–4 coprolites sharing nearly identical pollen assemblages and the same moa haplotype. Therefore, nearly half our entire sample (14–16 coprolites, including A10146, which has a distinct radiocarbon age) could be accounted for by just five individual birds over a period of about c. 6,500 years, indicating a slow, punctuated rate of accumulation within the deposit. Many of the other undated coprolites may also be from the same individuals. These findings suggest that a reasonable number of coprolites should be analysed in any future studies to ensure a number of different individuals are represented to overcome any potential sampling biases that may lead to over-representation of certain food taxa in the interpretation of overall diet. Analysing coprolites that are widely spaced within excavations, rather than adjacent specimens, or implementing random sample selection protocols, should also be considered as a way to reduce the chance of repeat sampling from single deposition events.

### Diet Proxy Biases and Limitations

Difficulties associated with interpreting diet from fecal studies have been well described in the literature (e.g. [59], [60]). Biased proportions of plant taxa in dung may be due to seasonal effects (e.g. pollen and seeds during the flowering and fruiting seasons), differential rates of digestion for different plant tissues, or analytical methods used. Some studies have aimed to quantify these biases by measuring dietary intake and comparing with the output (e.g. [61], [62]), although this approach has obvious limitations for extinct species.

Another way to overcome potential biases, and the approach we use here, is to combine multiple diet proxies. A dietary component of large, soft, easily digested leaves, for example, may be under-represented by macrofossil remains but will be detected by ancient DNA analysis. Analysis of the Euphrates Cave coprolites produced several instances where using multiple proxies assisted with the diet interpretation. For example, the abundance of wind- dispersed Poaceae pollen in many of the coprolites could have led to the interpretation that grasses formed an important part of upland moa diet. However, just a single DNA sequence from Poaceae was obtained from 199 clones (Fig. S4), indicating that although some grasses probably were eaten, much of the grass pollen may have been incidentally ingested while birds were feeding on herbs between flowering tussocks.

All three diet proxies (ancient DNA, macrofossils and pollen) were used on eight coprolites, and a summary of plant taxa identified in these samples using the different methods are shown in Fig. 4. Each proxy revealed plant taxa not detected by the others, supporting the conclusion of Jorgensen et al. [63] that these proxies are complimentary, and reinforcing that a multiproxy approach is necessary for gaining maximum paleodietary information from coprolite studies. Pollen assemblages included the largest number of plant taxa, but this is probably due to the presence of environmental pollen in addition to dietary taxa. Relatively few plant taxa were detected from macrofossils, due to the fine, highly digested nature of the coprolite matrix. There was no apparent bias in proxies towards any of the nine local habitat types identified by Druce et al. [38] (Fig. 5).

### Holocene Vegetation Change

Replacement of podocarp forests by a post-glacial spread of *Nothofagus* is a common pattern of vegetation change detected in sedimentary records across the South Island during the early-late Holocene [64]–[66]. However, despite the coprolites spanning a period of c. 6,500 cal. years, there is no evidence from the plant remains in the coprolites that would indicate major vegetation change in the vicinity of Garibaldi Plateau during this time. *Nothofagus* pollen is common in the coprolites, and suggests *Nothofagus* was the dominant forest type around Euphrates Cave by c. 7,300 cal. BP. Glacial refugia for *Nothofagus* are likely to have existed in the northwest region of the South Island [67], and so the surrounding areas may have been the earliest centres of *Nothofagus* recolonisation following glacial retreat. Certainly *Nothofagus* was established in nearby areas by the end of the Pleistocene [68]. A soil pollen core from the Garibaldi Range would provide useful information on local vegetation change during the Holocene, particularly for measuring changes in treeline boundaries. The latter would dictate whether grasslands may have occurred below the eastern buttress, and therefore have been more accessible to moa from the cave entrance. Based on current vegetation distributions, access to grassland from the cave would require walking approximately 600 m to the closest ridgeline leading to the plateau (a route currently used by deer).

### *Megalapteryx* Diet and Ecology

At least 67 plant taxa were identified from the Euphrates Cave coprolites by different techniques. Several of these have previously been reported from upland moa coprolites, e.g. *Nothofagus*, *Coprosma*, Cyperaceae [23], [24]. However, we also found several new records of likely food species, including *Phormium*, *Fuchsia* (both have nectar-rich flowers utilised by several extant bird species) and *Astelia*.

Druce et al. [38] defined nine major habitat-types occurring on the Garibaldi Range. All of these presently occur within one kilometre of the main entrance to Euphrates Cave, and plant taxa from each of these habitats were identified in the coprolites (Fig. 5).

Intact seeds, which may have been dispersed by upland moa, included species previously recorded from moa coprolites e.g. *Gaultheria*, Lobeliaceae (cf. *Pratia*), *Colobanthus*, and Cyperaceae [24]. The presence of intact *Fuchsia* seeds suggests moa were former dispersers for these plants too. Current seed dispersal relationships between New Zealand’s subalpine flora and fauna (both native and introduced) are poorly understood, but our data provide a baseline against which future studies on this topic could compare and identify post-settlement changes in seed dispersal dynamics.

Several of the plant taxa identified in the coprolites are regarded as palatable to mammalian herbivores (e.g. deer and goats), including broadleaf (*Griselinia*), *Pseudopanax, Fuchsia, Aristotelia* and *Coprosma* (cf. mamangi, *C. arborea*) [69], but these palatable taxa have been notably rare or absent in previous macrofossil analyses of moa gizzards and coprolites [19]–[25]. They were only detected by a multi-proxy approach through pollen and ancient DNA, which suggests they are readily digested and do not preserve as large identifiable fragments in the dung. Although we cannot determine which plants were preferred fodder, their presence in the coprolites indicates that they were palatable to upland moa, and raises the possibility that herbivory may potentially have resulted in reduced densities of these taxa in the past; an assumption underpinning the ‘podocarp regeneration-gap hypothesis’ [27]. Several of the plant taxa identified in the moa coprolites also exhibit putative defence structures suggested to have co-evolved against browsing [26], [27], [17], including wiry stems (common in *Coprosma, Myrsine,* rōhutu (*Neomyrtus*), bush lawyer (*Rubus*), and pōhuehue (*Muehlenbeckia*)) and turpines (grass tree (*Dracophyllum*)).

Due to its elevation, the Garibaldi Plateau receives a large amount of snowfall during winter, so there is likely to be a significant seasonal bias in the Euphrates Cave coprolites. The dominance of pollen from late spring-summer flowering plants suggests that most coprolites probably represent feeding during these seasons. For example, the pollen assemblage of A10164 is dominated by *Phormium*, which flowers from November to January. The significant amount of grass pollen in many of the coprolites, and the presence of *Fuchsia* pollen and seeds, also suggest feeding during the summer months. It is likely that upland moa moved from the subalpine zone into lower elevation forests during winter, as the extant flightless herbivorous takahe (*Porphyrio hochstetteri*) still does [70], [71].

The coprolites provide some evidence for recent changes in plant abundance and distribution since human settlement. *Fuchsia* and wineberry (*Aristotelia*) were identified from the coprolites using both DNA and pollen, yet were not recorded from the Garibaldi Range by Druce et al. [38]. Both taxa are highly palatable to introduced herbivores [69], [72] and can suffer severe local declines due to over-browsing [73]. Further evidence that the herbivory pressure exerted by introduced mammals on Garibaldi Plateau is greater than that exerted under the prehuman avian-dominated regime comes from the large number of plant taxa in the coprolites (34.3%) that are now largely restricted to trench and sinkhole walls (Fig. 5). Rather than reflecting a tendency for upland moa to feed around these holes, it is more likely that these sites are now refuges to a range of palatable plants, which once may have been more widespread in subalpine herbfields but are now heavily browsed by introduced mammalian herbivores.

Overall, the floral composition of the Euphrates Cave coprolites support previous interpretations of the feeding ecology of upland moa as highly generalist, comprising both browsing of trees and shrubs, and grazing of low herbs, in a broad range of habitats including forest, shrubland and grassland. Further work is required to broaden our knowledge of the diets of other moa species, however based on current understanding there seem to be significant similarities between different moa species. There is evidence that *Dinornis robustus* and *Pachyornis elephantopus*, like *Megalapteryx*, also had combined browsing and grazing feeding ecologies [20], [21], [24]. *Emeus* and *Euryapteryx* appear to have been browsers, particularly of soft leaves and fruit [20], [24] but there are relatively few samples of dietary remains from these moa taxa. The apparent overlap in feeding ecologies between moa, particularly *Megalapteryx, Dinornis* and *Pachyornis*, highlights a need for further study to clarify how niche partitioning occurred between sympatric moa species.

Our study confirms that, over a c. 6,500 year period around Euphrates Cave, upland moa were highly-generalist herbivores. Using ancient DNA analysis, pollen, and macrofossils, we identified at least 67 plant taxa in the coprolites, including the first evidence for moa having fed on nectar-rich flowers of New Zealand flax (*Phormium*) and tree fuchsia (*Fuchsia excorticata*). The identified taxa reveal that upland moa fed on both woody and herbaceous plants across a broad range of habitat types, and were potentially important seed dispersers for a range of plants. We also show that for coprolite studies multiproxy analysis of large sample sizes is necessary to overcome potential plant proxy and taphonomic biases, and gain the most complete picture of diets and habitats of extinct species.

## Supporting Information

Figure S1 **The 35 coprolites sampled in this study.**
(TIF)

Figure S2 **Alignment of control region sequences from upland moa (** ***Megalapteryx didinus*** **) showing the significant geographic haplotype variation, compared with sequences obtained from Euphrates Cave coprolites.**
(DOCX)

Figure S3 **Maximum clade credibility tree for moa control region sequences (Genbank accession numbers given) created using BEAST (HKY, MCMC chain length  = 10 million).** Moa species: red, *Dinornis novaezealandiae*; orange, *D. robustus*; yellow, *Megalapteryx didinus*; green, *Anomalopteryx didiformis*; cyan, *Emeus crassus*; blue, *Euryapteryx curtus*; purple, *Pachyornis elephantopus*; violet, *P. australis*. Note that *P. australis* is nested within *P. elephantopus* in some analyses [74] but separate in others [14]. The position of Euphrates Cave coprolite sequences are indicated by arrows. Branch labelling within *M. didinus* indicates geographic structuring. The Euphrates Cave coprolite sequences nest within the Nelson/West Coast clade.
(TIF)

Figure S4 **Alignment of rbcL clone sequences from Euphrates Cave upland moa (** ***Megalapteryx didinus*** **) coprolites, with identities and support values.**
(DOCX)

Figure S5 **Bayesian maximum credibility tree showing inferred relationships among plants documented in the Garibaldi range** [**38**] **and rbcL sequences isolated from upland moa (** ***Megalapteryx didinus*** **) coprolites.**
(PDF)

Table S1 **Pollen and spore count data for Euphrates Cave moa coprolites.** Values of 0.01 represent trace amounts observed while scanning pollen slide, but not encountered during actual count transects.
(DOCX)

Table S2 **Plant macrofossils and invertebrate remains from Euphrates Cave moa coprolites.**
(DOCX)
