# Supplementary figures and images for: High-Resolution Coproecology: Using Coprolites to Reconstruct the Habits and Habitats of New Zealand’s Extinct Upland Moa (Megalapteryx didinus)
Source: PLoS One. 2012 Jun 29;7(6):e40025. doi: 10.1371/journal.pone.0040025 (PMC3386916; doi:10.1371/journal.pone.0040025)

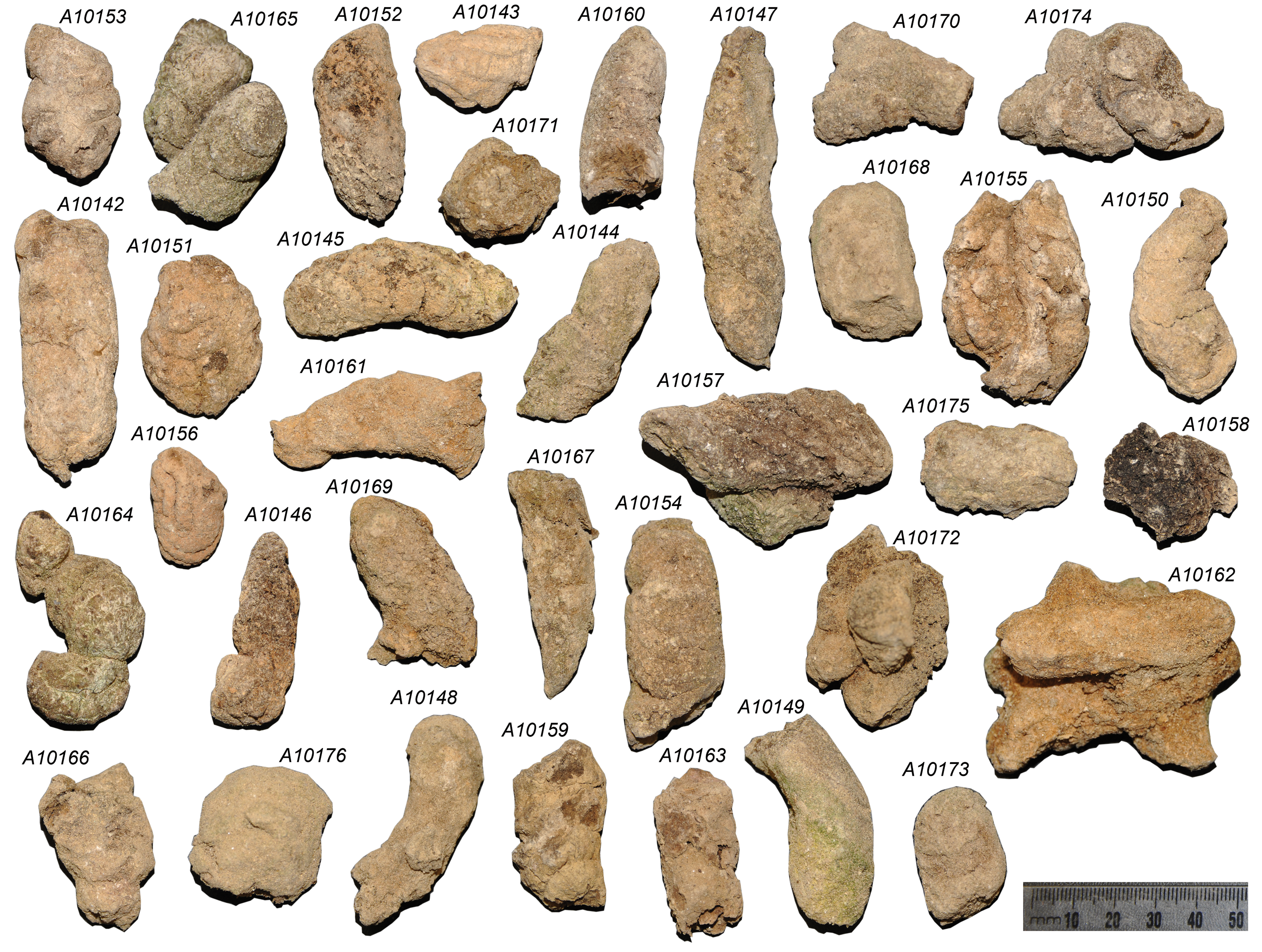

Supplement: Figure S1 — The 35 coprolites sampled in this study. (TIF) [file pone.0040025.s001.tif]

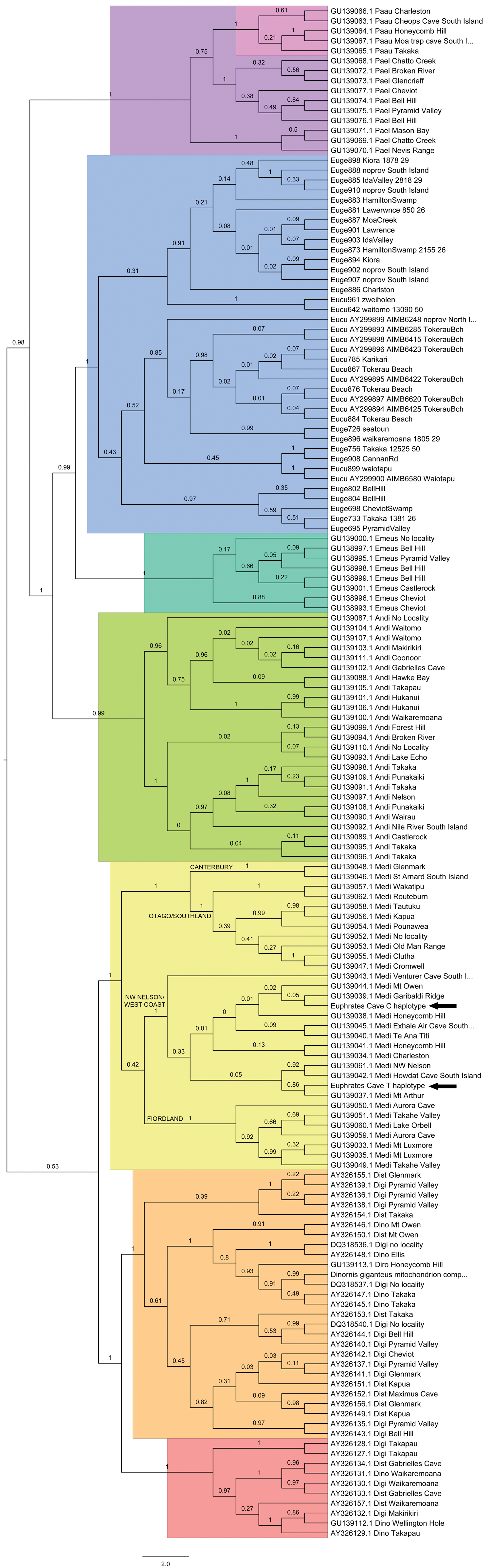

Supplement: Figure S3 — Maximum clade credibility tree for moa control region sequences (Genbank accession numbers given) created using BEAST (HKY, MCMC chain length = 10 million). Moa species: red, Dinornis novaezealandiae; orange, D. robustus; yellow, Megalapteryx didinus; green, Anomalopteryx didiformis; cyan, Emeus crassus; blue, Euryapteryx curtus; purple, Pachyornis elephantopus; violet, P. australis. Note that P. australis is nested within P. elephantopus in some analyses [74] but separate in others [14]. The position of Euphrates Cave coprolite sequences are indicated by arrows. Branch labelling within M. didinus indicates geographic structuring. The Euphrates Cave coprolite sequences nest within the Nelson/West Coast clade. (TIF) [file pone.0040025.s003.tif]
