# Supplementary material for: High-Resolution Coproecology: Using Coprolites to Reconstruct the Habits and Habitats of New Zealand’s Extinct Upland Moa (Megalapteryx didinus)
Source: PLoS One. 2012 Jun 29;7(6):e40025. doi: 10.1371/journal.pone.0040025 (PMC3386916; doi:10.1371/journal.pone.0040025)
Supplement: Figure S2 — Alignment of control region sequences from upland moa ( Megalapteryx didinus ) showing the significant geographic haplotype variation, compared with sequences obtained from Euphrates Cave coprolites. (DOCX) [file pone.0040025.s002.docx]

Moa_262 Forward> <Moa_294 Reverse

Consensus sequence GCGAAGACTGACTAGAAGCATAGATTTATAACCCGGACATAAATTTTACTCGTACTGTTCAAATCTCGC

**REFERENCE SEQUENCES**

NORTHWEST NELSON/WEST COAST

GU139037.1 *Megalapteryx* Mt Arthur ............................... (T haplotype)

GU139034.1 *Megalapteryx* Charleston ........................C...... (C haplotype)

CANTERBURY

GU139046.1 *Megalapteryx* St Arnaud ..........................C...C

GU139048.1 *Megalapteryx* Glenmark ......C.................C.C...C

FIORDLAND

GU139060.1 *Megalapteryx* Takahe Valley .....C..................C.....C

GU139059.1 *Megalapteryx* Aurora Cave ...A.C..................C.....C

OTAGO/SOUTHLAND

GU139054.1 *Megalapteryx* Pounawea ...A..C..............GCCC.....C

GU139053.1 *Megalapteryx* Old Man Range ......C..............GCCC.....C

GU139055.1 *Megalapteryx* Clutha ......C..............GCCC.C..TC

GU139062.1 *Megalapteryx* Routeburn .....C...............GCCC.....C

**COPROLITES**

A10144 Euphrates Cave ...............................

A10146 Euphrates Cave ...............................

A10147 Euphrates Cave ...............................

A10148 Euphrates Cave ...............................

A10150 Euphrates Cave ...............................

A10154 Euphrates Cave ...............................

A10155 Euphrates Cave ...............................

A10158 Euphrates Cave ...............................

A10160 Euphrates Cave ...............................

A10161 Euphrates Cave ...............................

A10162 Euphrates Cave ...............................

A10163 Euphrates Cave ...............................

A10166 Euphrates Cave ...............................

A10167 Euphrates Cave ...............................

A10168 Euphrates Cave ...............................

A10170 Euphrates Cave ...............................

A10171 Euphrates Cave ...............................

A10172 Euphrates Cave ...............................

A10173 Euphrates Cave ...............................

A10175 Euphrates Cave ...............................

A10176 Euphrates Cave ...............................

A10142 Euphrates Cave ........................C......

A10143 Euphrates Cave ........................C......

A10145 Euphrates Cave ........................C......

A10149 Euphrates Cave ........................C......

A10151 Euphrates Cave ........................C......

A10152 Euphrates Cave ........................C......

A10153 Euphrates Cave ........................C......

A10156 Euphrates Cave ........................C......

A10159 Euphrates Cave ........................C......

A10164 Euphrates Cave ........................C......

A10165 Euphrates Cave ........................C......

A10174 Euphrates Cave ........................C......
