# Supplementary material for: High-Resolution Coproecology: Using Coprolites to Reconstruct the Habits and Habitats of New Zealand’s Extinct Upland Moa (Megalapteryx didinus)
Source: PLoS One. 2012 Jun 29;7(6):e40025. doi: 10.1371/journal.pone.0040025 (PMC3386916; doi:10.1371/journal.pone.0040025)
Supplement: Figure S4 — Alignment of rbcL clone sequences from Euphrates Cave upland moa ( Megalapteryx didinus ) coprolites, with identities and support values. (DOCX) [file pone.0040025.s004.docx]

Coprolite

number

AACCYGGAGTTCCRCCTGAGGAAGCRGGGGCWGCRGTAGCTGCSGAATCTTCTACTGGTACATGGACAACTGTGTGGACCGATGGACTTACCAGT

10151 .G.TC..G.....G.....A.....A..A..T..A........G.............................T.....T............... Poaceae (1.00), st Danthonieae (0.99)

10174 ....C.....C..C.....A.....A..A..T..A.....G..A.........................................G........C Cyperaceae (1.00), st *Carex* (0.16)

10170 ....C.....C..T.....A.....A..A..T..A.....G..G.............................T.....T............... Cyperaceae (1.00), st *Desmoschoenus* (0.27)

10174 ....C.....C..C.....A.....A..A..T..A.....G..A.............................T.....T............... Cyperaceae (1.00), st *Uncinia* (0.27)

10174 ....C.....C..C.....A.....A..A..T..A.....G..A.............................T.....T............... "

10159 ...................A.....A.....Y..S........C......................................Y..R........C ?

10170 ....T........G...........G.....T..G........G...................................T...........T..C Fagales (0.09), *Nothofagus* (0.09)

10170 ....T........C...........G.....T..G........G.....C....................G....................G..C "

10176 ....C........G...........G.....T..G....A...G.....C................T...G........................ "

10151 ....C........G...........G.....T..G........G.....C................T...G........................ "

10176 ....C........G...........G.....T..G........G.....C................T...G........................ "

10170 ....C........G...........G.....T..G........G.....C....................G....................T..C "

10176 ....C........G...........G.....T..G........G.....C....................G.......................C "

10151 ....T........G...........G.....T..G........G.....C....................G........................ "

10151 ....C........G...........G.....T..G........G.....C....................G.....A.................. "

10151 ....C........G...........G.....T..G........G.....C....................G.....A.................. "

10151 ....C........GA..........G.....T..G........G.....C....................G........................ "

10151 ....C........G........C..G.....T..G........G.....C....................G........................ "

10158 ....C........G...........G.....T..G........G.....C.................G..G........................ "

10176 ....C........G...........G.....T.TG........G.....C....................G........................ "

10176 ....C........A...........G.....T..G........G.....C....................G........................ "

10151 ....C........G...........G.....T..G........G.....C....................G........................ "

10151 ....C........G...........G.....T..G........G.....C....................G........................ "

10151 ....C........G...........G.....T..G........G.....C....................G........................ "

10151 ....C........G...........G.....T..G........G.....C....................G........................ "

10151 ....C........G...........G.....T..G........G.....C....................G........................ "

10151 ....C........G...........G.....T..G........G.....C....................G........................ "

10151 ....C........G...........G.....T..G........G.....C....................G........................ "

10151 ....C........G...........G.....T..G........G.....C....................G........................ "

10151 ....C........G...........G.....T..G........G.....C....................G........................ "

10151 ....C........G...........G.....T..G........G.....C....................G........................ "

10151 ....C........G...........G.....T..G........G.....C....................G........................ "

10158 ....C........G...........G.....T..G........G.....C....................G........................ "

10158 ....C........G...........G.....T..G........G.....C....................G........................ "

10158 ....C........G...........G.....T..G........G.....C....................G........................ "

10158 ....C........G...........G.....T..G........G.....C....................G........................ "

10158 ....C........G...........G.....T..G........G.....C....................G........................ "

10158 ....C........G...........G.....T..G........G.....C....................G........................ "

10158 ....C........G...........G.....T..G........G.....C....................G........................ "

10158 ....C........G...........G.....T..G........G.....C....................G........................ "

10158 ....C........G...........G.....T..G........G.....C....................G........................ "

10158 ....C........G...........G.....T..G........G.....C....................G........................ "

10158 ....C........G...........G.....T..G........G.....C....................G........................ "

10158 ....C........G...........G.....T..G........G.....C....................G........................ "

10170 ....C........G...........G.....T..G........G.....C....................G........................ "

10170 ....C........G...........G.....T..G........G.....C....................G........................ "

10170 ....C........G...........G.....T..G........G.....C....................G........................ "

10170 ....C........G...........G.....T..G........G.....C....................G........................ "

10170 ....C........G...........G.....T..G........G.....C....................G........................ "

10170 ....C........G...........G.....T..G........G.....C....................G........................ "

10176 ....C........G...........G.....T..G........G.....C....................G........................ "

10176 ....C........G...........G.....T..G........G.....C....................G........................ "

10176 ....C........G...........G.....T..G........G.....C....................G........................ "

10176 ....C........G...........G.....T..G........G.....C....................G........................ "

10176 ....C........G...........G.....T..G........G.....C....................G........................ "

10176 ....C........G...........G.....T..G........G.....C....................G........................ "

10176 ....C........G...........G.....T..G........G.....C....................G........................ "

10176 ....C........G...........G.....T..G........C.......................T........................... st Orchidaceae (0.35)

10176 ....C........G...........G.....T..G........C...........A..............G........................ "

10143 ....C........G...........A.....T..G........T.........................................G......TCC Violaceae (1.00), st *Viola* (1.00)

10176 ....C........G...........G.....A..A........T........................G................G......... Cunoniaceae (0.33), st *Weinmannia* (0.33)

10145 ....G........G...........C.....A..G........T..................................................C ?

10143 ....T........G...........C.....A..A....T...T..................................................C Oxalidales (0.82), Oxalidaceae (0.11)

10143 ....T........G...........C.....A..G........T...................................T.....G......... Oxalidales (0.82), Elaeocarpaceae (0.24)

10143 ....T........G...........C.....A..G........T....................A....................G......... "

10143 ....T........G...........C.....A..G........T....................A....................G......... "

10145 ....T........G...........C.A...A..G........T.........................................G......... "

10145 ....T........C...........C.....A..G........T.........................................G......... Oxalidales (0.82), Oxalidaceae (0.11)

10145 ....T........C...........C.....A..G........T.........................................G......... "

10143 ....T........G...........C.....A..G........T.........................................G......... Oxalidales (0.82), Elaeocarpaceae (0.24)

10143 ....T........G...........C.....A..G........T.........................................G......... "

10143 ....T........G...........C.....A..G........T.........................................G......... "

10143 ....T........G...........C.....A..G........T.........................................G......... "

10143 ....T........G...........C.....A..G........T.........................................G......... "

10143 ....T........G...........C.....A..G........T.........................................G......... "

10143 ....T........G...........C.....A..G........T.........................................G......... "

10143 ....T........G...........C.....A..G........T.........................................G......... "

10143 ....T........G...........C.....A..G........T.........................................G......... "

10145 ....T........G...........C.....A..G........T.........................................G......... "

10145 ....T........G...........C.....A..G........T.........................................G......... "

10145 ....T........G...........C.....A..G........T.........................................G......... "

10145 ....T........G...........C.....A..G........T.........................................G......... "

10145 ....T........G...........C.....A..G........T.........................................G......... "

10145 ....T........G...........C.....A..G........T.........................................G......... "

10145 ....T........G...........C.....A..G........T.........................................G......... "

10143 ....T........G...........C.....A..A........T................................................... "

10176 ....T........C...........C.....A..A........T..........................G........................ "

10143 ....T........C...........C.....A..A........T................................................... "

10174 ....T........C...........C.....A..A........T................................................... "

10143 ....T........C...........C.....A..A........T.........................................G........C "

10143 ....T........C...........C.....A..A........T.........................................G........C "

10143 ....T........C...........C.....A..A....Y...T.........................................G......... "

10143 ....T........C...........C.....A..A....Y...T.........................................G......... "

10145 ....T........C...........C.....A..A........T................T........................G......... "

10145 ....T........C...........C.....A..A......A.T.........................................G......... "

10145 ....T........C...........C.....A..A......A.T.........................................G......... "

10143 ....T........C...........C.....A..A........T.........................................G......... "

10145 ....T........C...........C.....A..A........T.........................................G......... "

10145 ....T........C...........C.....A..A........T.........................................G......... "

10145 ....T........C...........C.....A..A........T.........................................G......... "

10145 ....T........C...........C.....A..A........T.........................................G......... "

10145 ....T........C...........C.....A..A........T.........................................G......... "

10145 ....T........C...........C.....A..A........T.........................................G......... "

10151 ....T........C...........C.....A..A........T.........................................G......... "

10151 ....T........C...........C.....A..A........T.........................................G......... "

10159 ....T........C...........C.....A..A........T.........................................G......... "

10159 ....T........C...........C.....A..A........T.........................................G......... "

10159 ....T........C...........C.....A..A........T.........................................G......... "

10170 ....T........C...........C.....A..A........T.........................................G......... "

10174 ....T........C...........C.....A..A........T.........................................G......... "

10174 ....T........C...........C.....A..A........T.........................................G......... "

10174 ....T........C...........C.....A..A........T.........................................G......... "

10174 ....T........C...........C.....A..A........T.........................................G......... "

10174 ....T........C...........C.....A..A........T.........................................G......... "

10174 ....T........C...........C.....A..A........T.........................................G......... "

10174 ....T........C...........C.....A..A........T.........................................G......... "

10143 ....T..C.....G...........A.....T..G........T..................................................C Fagales (0.09), *Nothofagus* (0.09)

10158 ....T........G...........A.....G..G........T................................................... "

10143 ....T..C.....G...........A.....G..G........G.............................A.....T..C............ Rosaceae (1.00), st *Acaena* (1.00)

10143 ....T..C.....G...........A.....G..G........G.............................A.....T..C............ "

10159 ....T..C.....G...........A.....G..G........G.............................A.....T..C............ "

10159 .......K..C..G...........A.....GA.G........G.............................A.....T..C............ "

10170 ....C........A..G..A.....A.....C..G........C..G..........................A.....G.............A. Rubiaceae (0.88), st *Coprosma arborea* (0.96)

10170 ....A........A..G..A.....A.....C..G........C..G..........................A.....G............... Rubiaceae (0.88), *Coprosma/Nertera* (0.99)

10174 ....C........A..G..A.....A.....C..G........C..G...C......................A.....G............... "

10170 ....C........A..G..A.....A.....C..G........C..G..........................A.....G............... "

10174 ....C........A..G..A.....A.....C..G........C..G..........................A.....G............... "

10174 ....C........A..G..A.....A.....C..G........C..G..........................A.....G............... "

10176 ....C........A..G..A.....A.....C..G........C..G..........................A.....G............... "

10158 ....T........T.....A.....A.....G..A........C.............................A.....T..C............ Boraginaceae (0.57), st *Myosotis* (0.57)

10159 ....G........A.....A.....G.....T..T........C...........A......................................C Ranunculaceae (1.00), st *Ranunculus* (1.00)

10159 ....G........A.....A.....G.....T..T........C...........A......................................C "

10159 ....G........A.....A.....G.....T..T........C...........A......................................C "

10159 ....G........A.....A.....G.....T..T........C...........A......................................C "

10159 ....G........A.....A.....G.....T..C........T......................................C..G........C Brassicaceae (1.00), st *Cardamine* (0.93)

10170 ....A........A.....A.....A.....C..G.....G..G...................................T...........T..C Ericaceae (0.73), *Gaultheria* (1.00)

10170 ....A........A.....A.....A.....C..G.....G..G...................................T...........T..C "

10170 ....A........A.....A.....A.....C..G.....G..G...................................T...........T..C "

10151 ....G........G.....A.....A.....C..G............................................Y...........T..C Ericaceae (0.73), *Dracophyllum* (0.41)

10158 ....G........A...........A.....C..T........T.........................................G......... Coriariaceae (1.00), st *Coriaria* (1.00)

10170 ....T........G...........A.....T..A........C.................C.......................G......... Onagraceae (0.85), st *Fuchsia* (0.40)

10170 ....T........G...........A.....T..A........C.................C.......................G........C Onagraceae (0.85), st *Fuchsia* (0.44)

10170 ....T........G...........A.....T..A........C.................C.......................G........C "

10170 ....T........G...........A.....T..A........C.................C.......................G........C "

10170 ....T........G...........A.....T..A........C.................C.......................G........C "

10170 ....T........G...........A.....T..A........C.................C.......................G........C "

10170 ....T........G...........A.....T..A........C.................C.......................G........C "

10170 ....C........G..C..A.....A.....T..A........C.........................................G......... Piperaceae (0.46), st *Macropiper* (0.46)

10170 ....C........G..C..A.....A.....T..A........C.................C.......................G........C "

10170 ....T........A.....A.....A...T.T..A........C..........................C....................T..C Primulaceae (0.42), *Myrsine* (0.63)

10170 ....A........A..G..A.....A.....T..A........C..........................C....................T..C "

10170 ....A........A.....A.....A.....T..G........C..........................C....................T..C "

10170 ....A........A.....A.....A.....T..A........C..........................C....................T..C "

10170 ....C........G..C..A.....A.....T.TA........C..........................C....................G..C "

10170 ....T........G..C..A.....A.....T..A........C..........................C....................T..C "

10170 ....A........G..C..A.....A.....T..A........C..........................C....................T..C "

10170 ....C........G..C..A.....A.....T..A........C..........................C.......................C Piperaceae (0.39), st *Macropiper* (0.39)

10170 ....C........G..C..A.....A.....T..A........C..........................C.......................C "

10170 ....C........G..C..A.....A.....T..A........C..........................C.......................C "

10170 ....C........G..C..A.....A...T.T..A........C..........................C....................T..C Primulaceae (0.42), *Myrsine* (0.63)

10170 ....C........G..C..A.....A.....T..A........C..........................C....................T..C "

10170 ....C........G..C..A.....A.....T..A........C..........................C....................T..C "

10170 ....C........G..C..A.....A.....T..A........C..........................C....................T..C "

10170 ....C........G..C..A.....A.....T..A........C..........................C....................T..C "

10170 ....C........G..C..A.....A.....T..A........C..........................C....................T..C "

10170 ....C........G..C..A.....A.....T..A........C..........................C....................T..C "

10170 ....C........G..C..A.....A.....T..A........C..........................C....................T..C "

10170 ....C........G..C..A.....A.....T..A........C..........................C....................T..C "

10170 ....C........G..C..A.....A.....T..A........C..........................C....................T..C "

10170 ....C........G..C..A.....A.....T..A........C..........................C....................T..C "

10170 ....C........G..C..A.....A.....T..A........C..........................C....................T..C "

10170 ....C........G..C..A.....A.....T..A........C..........................C....................T..C "

10174 ....C........G..C..A.....A.....T..A........C..........................C....................T..C "

10170 ....C........A..G..A.....A.....C..G........C..G................................T...........T..C Gentianales (0.31), *Galium/Coprosma/Nertera* (0.88)

10176 ....C........A.....A.....A.....C..G........C...............................................T..C Gentianaceae (0.89)

10176 ....C........A.....A.....A.....C..G........C...............................................T..C "

10176 ....C........A.....A.....A.....C..G.....A..C........G.........................................C st Plantaginaceae (0.97), st *Veronica* (0.97)

10176 ....G........A.....A.....A..C..T..G.....A..C........G.........................................C Plantaginaceae (0.50), *Veronica* (1.00)

10176 ....G........A.....A.....A..C..T..G.....A..C........G.........................................C "

10176 ....G........A.....A.....A..C..T..G.....A..C........G.......................................... "

10143 ....T........A.....A.....G.....C..G........C...................................T.....G......... ?

10174 ....A........A.....A.....G.....C..G........C.........................................G......... ?

10174 ....G........A.....A.....A.....C..GA.......G.............................T.....T............... Ericaceae (0.73)

10174 ....C........A..G..A.....A.....C..G........G...................................T............... "

10174 ....G........A.....A.....A.....C..G........G...................................T.....G......... "

10159 ....T..............A.....A.....C..A........C...................................Y............... Stylidiaceae (0.52), st *Forstera/Phyllachne* (0.52)

10159 ....T........G.....A.....A.....C..A........C......................................C..G........C Loganiaceae (0.48), st *Mitrasacme* (0.48)

10145 ....T........T.....A.....A.....G..A........C..................................................C Boraginaceae (0.57), st *Myosotis* (0.81)

10159 ....T........G.....A.....A.....C..A........C..................................................C Asteraceae (0.91), st *Taraxacum* (0.29)

10159 ....T........G.....A.....A.....C..A........C...............................................G..C "

10159 ....C........A.....A.....A.....T..C........T..................................................C Brassicaceae (1.00), st *Cardamine* (0.85)

10170 ....T........A.....A.....A.....T..C........C..................................................C Araliaceae (0.89), *Pseudopanax* (1.00)

10174 ....C.....C..C.....A.....G.....C..G........C..................................................C Paracryphiaceae (0.48), st *Quintinia* (0.48)

10159 ....T........C...........G.....C..G........C..................................................C Grisilineaceae (0.06), *Griselinia* (0.06)

10143 ....T........A.....A.....G.....C..G........C..................................................C "

10145 ....T........A.....A.....G.....C..G........C..................................................C "

10145 ....T........A.....A.....G.....C..G........C..................................................C "

10174 ....T........A.....A.....G.....C..G........C..................................................C "

10174 ....T........A.....A.....G.....C..G........C..................................................C "
