# Supplementary material for: High-Resolution Coproecology: Using Coprolites to Reconstruct the Habits and Habitats of New Zealand’s Extinct Upland Moa (Megalapteryx didinus)
Source: PLoS One. 2012 Jun 29;7(6):e40025. doi: 10.1371/journal.pone.0040025 (PMC3386916; doi:10.1371/journal.pone.0040025)
Supplement: Figure S5 — Bayesian maximum credibility tree showing inferred relationships among plants documented in the Garibaldi range [38] and rbcL sequences isolated from upland moa ( Megalapteryx didinus ) coprolites. (PDF) [file pone.0040025.s005.pdf]

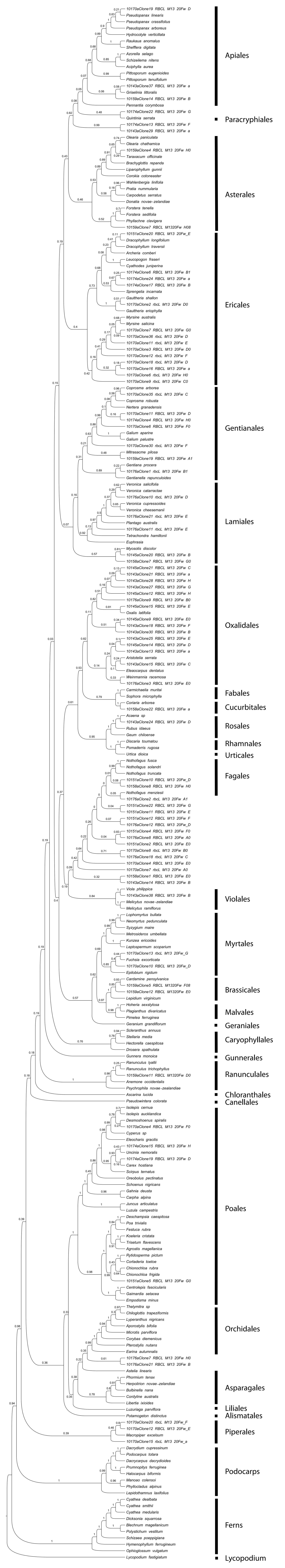

Apiales
Paracryphiales
Asterales
Ericales
Gentianales
Lamiales
Oxalidales
Fabales
Cucurbitales
Rosales
Rhamnales
Urticales
Fagales
Violales
Myrtales
Brassicales
Malvales
Geraniales
Caryophyllales
Gunnerales
Ranunculales
Chloranthales
Canellales
Poales
Orchidales
Asparagales
Liliales
Alismatales
Piperales
Podocarps
Ferns
Lycopodium
