# Supplementary material for: High-Resolution Coproecology: Using Coprolites to Reconstruct the Habits and Habitats of New Zealand’s Extinct Upland Moa (Megalapteryx didinus)
Source: PLoS One. 2012 Jun 29;7(6):e40025. doi: 10.1371/journal.pone.0040025 (PMC3386916; doi:10.1371/journal.pone.0040025)
Supplement: Table S1 — Pollen and spore count data for Euphrates Cave moa coprolites. Values of 0.01 represent trace amounts observed while scanning pollen slide, but not encountered during actual count transects. (DOCX) [file pone.0040025.s006.docx]

| Sample | Sample mass (g) | Spike added | Spike count | Counted mass (g) | *Dacrydium cupressinum* | Fuscospora | *Nothofagus menziesii* | *Prumnopitys ferruginea* | *Prumnopitys taxifolia* | *Podocarpus* | *Halocarpus* | *Phyllocladus* | *Metrosideros* |
| --- | --- | --- | --- | --- | --- | --- | --- | --- | --- | --- | --- | --- | --- |
| A10142 | 0.5 | 37166 | 301 | 0.0040494 |  | 17 | 3 | 3 | 7 | 1 |  | 1 |  |
| A10143 | 0.54 | 37166 | 62 | 0.0009008 | 1 | 7 | 6 |  | 1 |  |  |  |  |
| A10144 | 0.42 | 37166 | 341 | 0.0038535 | 4 | 24 | 10 | 3 |  |  |  |  |  |
| A10145 | 0.51 | 37166 | 109 | 0.0014957 | 1 | 10 | 6 | 1 | 2 |  |  |  |  |
| A10146 | 0.5 | 37166 | 129 | 0.0017355 | 1 | 7 |  |  | 2 |  |  |  |  |
| A10147 | 0.44 | 37166 | 204 | 0.0024151 | 1 | 13 | 5 | 2 |  |  |  | 2 |  |
| A10148 | 0.38 | 37166 | 96 | 0.0009815 | 1 | 8 | 6 | 3 |  |  |  | 1 |  |
| A10149 | 0.44 | 37166 | 28 | 0.0003315 | 2 | 2 |  | 1 | 1 |  |  |  |  |
| A10150 | 0.39 | 37166 | 57 | 0.0005981 |  | 7 | 2 | 1 |  |  |  |  |  |
| A10151 | 0.53 | 37166 | 533 | 0.0076008 | 1 | 15 | 7 | 3 | 7 | 2 |  |  |  |
| A10152 | 0.46 | 37166 | 56 | 0.0006931 | 3 | 7 | 5 |  |  |  |  |  |  |
| A10153 | 0.52 | 37166 | 318 | 0.0044492 | 1 | 13 | 2 | 4 | 5 | 2 |  | 1 |  |
| A10154 | 0.35 | 37166 | 139 | 0.001309 | 2 | 16 | 9 |  | 0.01 |  |  |  |  |
| A10155 | 0.4 | 37166 | 14 | 0.0001507 |  | 2 | 1 |  |  | 0.01 |  |  |  |
| A10156 | 0.54 | 37166 | 13 | 0.0001889 |  | 5 | 1 | 1 | 2 |  |  |  |  |
| A10157 | 0.28 | 37166 | 60 | 0.000452 | 1 | 17 | 6 | 1 | 3 | 2 |  | 0.01 |  |
| A10158 | 0.39 | 37166 | 269 | 0.0028227 | 3 | 35 | 7 | 1 | 3 | 1 |  | 4 | 1 |
| A10159 | 0.45 | 37166 | 53 | 0.0006417 | 0.01 | 6 | 3 |  | 0.01 |  |  |  |  |
| A10160 | 0.08 | 37166 | 1889 | 0.0040661 | 4 | 50 | 12 | 3 | 2 |  |  | 1 | 1 |
| A10161 | 0.32 | 37166 | 35 | 0.0003014 | 1 | 9 |  |  | 1 | 0.01 | 1 |  |  |
| A10162 | 0.41 | 37166 | 36 | 0.0003971 | 2 | 18 | 3 |  | 2 | 0.01 |  | 2 |  |
| A10163 | 0.21 | 37166 | 52 | 0.0002938 | 0.01 | 2 | 1 |  | 0.01 |  |  |  |  |
| A10164 | 0.62 | 37166 | 28 | 0.0004671 | 0.01 | 1 |  |  | 1 |  |  |  |  |
| A10165 | 0.46 | 37166 | 247 | 0.0030571 | 7 | 14 |  | 1 | 5 | 3 |  | 1 | 1 |
| A10166 | 0.11 | 37166 | 370 | 0.0010951 | 1 | 15 | 6 | 2 |  |  |  |  |  |
| A10167 | 0.12 | 37166 | 57 | 0.000184 | 1 | 3 | 1 |  | 1 | 0.01 |  | 0.01 |  |
| A10168 | 0.38 | 37166 | 48 | 0.0004908 | 5 | 29 | 7 | 2 | 8 | 4 |  | 6 |  |
| A10169 | 0.47 | 37166 | 30 | 0.0003794 |  | 7 | 0.01 | 0.01 | 2 | 2 |  |  |  |
| A10170 | 0.25 | 37166 | 220 | 0.0014798 | 2 | 37 | 7 |  | 7 | 1 | 1 | 4 | 1 |
| A10171 | 0.24 | 37166 | 321 | 0.0020729 | 0.01 | 21 | 7 |  | 0.01 | 1 |  | 2 |  |
| A10172 | 0.36 | 37166 | 55 | 0.0005327 | 4 | 12 | 2 | 2 | 0.01 |  |  |  |  |
| A10173 | 0.46 | 37166 | 100 | 0.0012377 | 1 | 25 | 6 |  |  |  |  |  |  |
| A10174 | 0.41 | 37166 | 76 | 0.0008384 | 6 | 20 | 6 |  | 1 | 1 |  | 2 |  |
| A10175 | 0.4 | 37166 | 71 | 0.0007641 | 1 | 12 | 2 |  |  | 1 |  |  |  |
| A10176 | 0.36 | 37166 | 70 | 0.000678 | 1 | 10 | 2 |  | 0.01 | 1 |  | 0.01 |  |

| Sample | *Pseudopanax* undiff. | *Pseudopanax colensoi* | *Fuchsia excorticata* | *Ascarina lucida* | *Aristotelia-*type | *Griselinia* | *Myrsine* | *Coprosma* | *Neomyrtus-*type | *Parahebe* | Ericaceae | *Pimelea* | *Kelleria* | *Rubus* |
| --- | --- | --- | --- | --- | --- | --- | --- | --- | --- | --- | --- | --- | --- | --- |
| A10142 |  |  | 0.01 |  |  |  | 1 | 18 |  |  |  |  |  |  |
| A10143 |  |  | 14 |  |  |  |  | 1 |  |  |  |  |  |  |
| A10144 |  |  |  |  |  |  | 1 |  |  |  |  |  |  |  |
| A10145 |  |  | 13 |  |  |  |  |  |  |  |  |  |  |  |
| A10146 |  |  |  |  |  |  |  | 6 |  |  |  |  |  |  |
| A10147 |  | 1 |  |  |  |  | 1 |  |  |  |  |  |  |  |
| A10148 |  |  |  |  |  |  |  |  |  |  |  |  |  |  |
| A10149 |  |  |  |  |  |  |  |  |  |  |  |  |  |  |
| A10150 |  |  |  |  |  |  |  | 0.01 |  |  | 0.01 |  |  |  |
| A10151 |  |  |  |  |  |  |  | 14 |  |  | 5 |  |  |  |
| A10152 |  |  | 8 |  |  |  |  | 3 |  |  |  |  |  |  |
| A10153 |  |  |  |  |  |  |  | 23 |  | 1 |  |  |  |  |
| A10154 | 1 |  |  |  |  |  |  |  |  |  | 22 |  |  |  |
| A10155 |  |  |  |  |  |  |  | 1 | 1 |  | 0.01 |  |  |  |
| A10156 |  |  | 1 |  |  |  |  | 0.01 |  |  |  |  |  |  |
| A10157 |  |  |  |  |  |  |  | 1 |  |  |  |  |  |  |
| A10158 |  | 39 |  |  |  |  |  | 2 |  |  | 1 |  |  |  |
| A10159 |  |  |  |  |  |  |  |  |  |  |  | 1 |  |  |
| A10160 |  |  |  |  |  |  | 3 | 1 | 3 |  |  |  |  |  |
| A10161 |  | 1 | 1 |  |  |  |  |  |  |  |  |  |  |  |
| A10162 |  | 1 |  |  |  |  |  |  | 1 |  |  |  |  |  |
| A10163 |  | 1 |  |  |  |  |  | 0.01 |  |  | 2 |  |  |  |
| A10164 |  | 1 | 0.01 |  |  |  |  |  |  |  |  |  |  |  |
| A10165 | 4 | 2 |  |  |  | 1 | 1 | 7 |  |  | 14 |  |  |  |
| A10166 |  |  |  |  |  |  |  |  |  |  |  |  |  |  |
| A10167 |  |  | 0.01 |  |  |  |  |  |  |  |  |  |  |  |
| A10168 |  | 1 |  |  | 1 |  |  |  |  |  |  |  |  |  |
| A10169 |  |  |  |  |  |  |  | 1 |  |  |  |  |  |  |
| A10170 |  |  |  | 1 |  |  |  |  | 1 |  |  |  | 1 | 1 |
| A10171 | 0.01 |  |  | 0.01 |  |  |  | 1 | 1 |  | 0.01 |  |  |  |
| A10172 |  |  | 0.01 |  |  |  |  |  |  |  |  |  |  |  |
| A10173 |  |  |  |  |  |  |  | 1 | 1 |  | 1 |  |  |  |
| A10174 |  |  |  |  |  |  |  | 3 |  |  |  |  |  |  |
| A10175 |  |  |  |  |  |  |  |  |  |  |  |  |  |  |
| A10176 | 1 |  |  |  |  |  |  |  |  |  | 1 |  |  |  |

| Sample | *Muehlenbeckia* | Asteraceae | *Acaena* | Caryophyllaceae | Brassicaceae | *Epilobium* | *Gentiana* | *Drosera* | Lactuceae | *Plantago* | *Myosotis* | *Myriophyllum-*type | *Donatia* |
| --- | --- | --- | --- | --- | --- | --- | --- | --- | --- | --- | --- | --- | --- |
| A10142 |  | 38 |  |  | 13 | 1 | 1 |  |  |  | 3 |  |  |
| A10143 |  | 49 | 62 | 1 | 12 |  |  |  |  | 6 | 3 |  |  |
| A10144 |  | 4 | 1 | 1 | 1 |  | 3 |  |  |  | 2 |  |  |
| A10145 |  | 34 | 60 |  | 9 | 1 |  |  |  | 8 | 4 |  |  |
| A10146 |  |  |  | 1 |  |  |  |  |  | 3 | 3 |  |  |
| A10147 |  | 3 | 1 | 1 |  | 1 | 4 |  |  |  |  |  |  |
| A10148 |  | 1 | 1 |  | 3 | 1 | 34 |  | 2 | 5 | 2 |  |  |
| A10149 |  | 2 |  |  |  |  |  |  |  |  | 4 |  |  |
| A10150 |  | 26 | 4 |  | 4 |  | 3 | 3 | 6 |  | 2 |  |  |
| A10151 |  | 44 |  |  | 11 | 1 | 1 | 1 |  |  | 4 |  |  |
| A10152 |  | 26 | 51 | 1 | 5 |  |  |  | 1 | 8 | 5 |  |  |
| A10153 | 2 | 40 |  |  | 5 | 1 | 1 |  |  |  |  |  |  |
| A10154 |  | 14 |  |  | 3 |  | 3 |  | 2 |  | 6 |  |  |
| A10155 |  | 30 | 2 | 0.01 | 9 | 0.01 | 3 |  | 2 | 2 | 1 |  |  |
| A10156 |  |  |  | 0.01 | 6 | 1 | 2 |  | 4 |  | 6 |  |  |
| A10157 |  | 3 | 1 |  |  |  | 6 |  | 0.01 |  | 19 |  |  |
| A10158 |  | 20 |  | 1 |  |  |  |  |  |  | 2 |  |  |
| A10159 |  | 109 | 0.01 | 1 | 25 | 3 |  |  | 2 | 1 | 11 |  |  |
| A10160 |  | 10 |  | 1 | 2 |  | 1 |  |  | 7 | 4 |  |  |
| A10161 |  | 26 | 2 |  | 1 |  | 6 | 4 | 9 | 1 | 1 |  |  |
| A10162 |  | 5 | 1 | 0.01 | 7 |  | 39 | 0.01 | 20 | 0.01 | 3 |  |  |
| A10163 |  | 3 | 3 | 0.01 |  |  |  |  |  | 0.01 | 1 |  |  |
| A10164 |  | 9 |  | 0.01 | 4 |  | 1 |  |  |  | 6 |  |  |
| A10165 |  | 31 |  |  | 11 |  |  |  |  | 6 | 5 |  |  |
| A10166 |  | 3 | 1 | 0.01 | 4 | 1 | 27 |  | 1 |  | 9 |  |  |
| A10167 |  | 9 | 0.01 |  |  |  | 4 |  | 0.01 | 3 | 2 |  |  |
| A10168 |  | 4 |  |  |  | 1 | 17 |  | 3 | 2 | 5 |  |  |
| A10169 |  | 19 |  |  | 2 | 0.01 | 4 | 2 | 10 | 1 | 1 |  |  |
| A10170 |  | 5 |  |  |  |  | 23 |  | 1 |  | 1 |  |  |
| A10171 |  | 5 | 1 | 0.01 | 6 |  | 27 |  |  | 1 | 1 |  |  |
| A10172 |  | 15 | 3 |  | 5 |  | 44 |  | 21 |  | 11 |  |  |
| A10173 |  | 18 |  |  | 2 |  | 1 |  |  | 1 |  |  |  |
| A10174 |  | 12 | 1 |  | 6 | 3 |  |  | 1 | 8 | 22 |  |  |
| A10175 |  | 15 |  | 1 | 9 |  | 50 |  | 19 |  | 10 | 1 | 1 |
| A10176 |  | 9 | 1 |  | 4 | 0.01 | 44 |  |  |  | 14 |  | 1 |

| Sample | *Ranunculus* | Apiaceae | *Anisotome* | Poaceae | Cyperaceae | *Astelia* | *Bulbinella* | *Phormium* | *Cyathea colensoi* | *Hymenophyllum* | Monolete fern spores | *Ophioglossum* |
| --- | --- | --- | --- | --- | --- | --- | --- | --- | --- | --- | --- | --- |
| A10142 | 5 | 74 |  | 29 |  | 21 |  |  | 4 | 0.01 | 16 |  |
| A10143 |  |  |  | 68 |  | 119 |  | 31 | 4 |  | 45 | 7 |
| A10144 |  |  |  | 100 | 12 |  |  |  | 68 |  | 60 | 2 |
| A10145 |  |  |  | 35 | 1 | 63 |  | 10 |  | 1 | 58 | 13 |
| A10146 | 7 | 6 |  | 2 |  | 253 |  |  | 1 |  | 3 |  |
| A10147 | 1 |  |  | 119 | 5 |  |  |  | 52 |  | 42 | 4 |
| A10148 |  |  |  | 190 | 1 |  | 2 |  | 5 |  | 11 | 1 |
| A10149 |  | 1 |  | 71 | 187 |  |  | 6 |  | 1 |  |  |
| A10150 | 2 | 4 |  | 237 |  |  |  |  | 2 |  | 7 |  |
| A10151 | 9 | 72 |  | 29 | 1 | 14 |  |  |  |  | 15 |  |
| A10152 |  |  |  | 50 | 1 | 124 |  | 20 |  | 2 | 40 | 8 |
| A10153 | 13 | 65 |  | 30 | 4 | 22 | 1 |  | 1 |  | 11 | 1 |
| A10154 | 1 |  |  | 193 | 8 | 5 |  | 5 | 2 | 1 | 5 | 2 |
| A10155 | 2 |  | 18 | 184 | 16 | 12 | 0.01 | 5 |  |  | 2 |  |
| A10156 | 1 | 1 |  | 226 |  | 9 | 1 |  |  |  | 7 | 1 |
| A10157 | 1 |  |  | 186 | 3 | 10 |  | 3 |  |  | 4 | 3 |
| A10158 | 10 | 38 |  | 6 | 30 |  |  | 2 | 2 |  | 2 |  |
| A10159 |  | 10 |  | 65 | 2 |  | 0.01 |  | 0.01 |  | 89 |  |
| A10160 |  |  |  | 77 | 8 |  |  | 1 | 54 |  | 72 | 2 |
| A10161 | 6 | 1 |  | 185 |  |  |  | 1 | 4 |  | 9 |  |
| A10162 |  | 1 |  | 167 | 3 |  |  |  | 0.01 |  | 21 |  |
| A10163 | 0.01 | 3 |  | 36 | 183 | 23 | 5 | 1 | 0.01 | 8 | 1 |  |
| A10164 | 2 | 2 | 4 | 20 | 1 | 2 | 0.01 | 237 | 2 |  | 10 |  |
| A10165 | 4 | 73 | 3 | 27 | 5 | 26 |  |  |  | 1 | 13 |  |
| A10166 |  |  |  | 175 | 1 |  | 2 |  | 4 |  | 24 | 2 |
| A10167 | 5 | 0.01 |  | 270 |  | 3 |  |  | 0.01 |  | 8 |  |
| A10168 | 2 |  |  | 51 | 6 |  |  |  | 131 |  | 33 |  |
| A10169 | 5 | 1 |  | 278 |  |  |  | 1 | 0.01 |  | 8 |  |
| A10170 | 1 |  |  | 51 | 6 | 1 |  | 1 | 98 |  | 35 | 4 |
| A10171 |  | 0.01 |  | 86 | 8 |  |  |  | 1 | 1 | 85 |  |
| A10172 | 3 | 0.01 |  | 168 | 2 | 1 |  |  | 0.01 |  | 26 |  |
| A10173 | 3 | 41 |  | 10 | 19 | 99 |  |  | 1 | 10 | 1 |  |
| A10174 | 4 |  |  | 108 | 58 | 1 |  |  |  |  | 11 | 0.01 |
| A10175 | 2 | 0.01 |  | 189 | 1 | 1 |  |  | 1 |  | 24 |  |
| A10176 | 1 |  |  | 215 | 1 |  | 1 |  | 1 |  | 10 | 4 |

| Sample | *Lycopodium australinum* | *Lycopodium scarosum* | *Anthoceros* | Undeterminable | Total count | Pollen density (grains/g) | *Sporormiella* |
| --- | --- | --- | --- | --- | --- | --- | --- |
| A10142 |  |  |  | 14 | 270 | 66,677 | 1 |
| A10143 |  |  |  | 24 | 461 | 511,754 |  |
| A10144 |  |  |  | 22 | 318 | 82,522 | 1 |
| A10145 |  |  |  | 16 | 346 | 231,326 |  |
| A10146 |  |  |  | 6 | 301 | 173,441 |  |
| A10147 |  |  |  | 13 | 271 | 112,210 |  |
| A10148 |  |  |  | 14 | 292 | 297,491 |  |
| A10149 |  |  |  | 11 | 289 | 871,832 |  |
| A10150 |  |  |  | 5 | 315 | 526,644 |  |
| A10151 |  |  |  | 26 | 282 | 37,102 | 1 |
| A10152 |  |  |  | 18 | 386 | 556,913 |  |
| A10153 |  |  |  | 18 | 267 | 60,010 |  |
| A10154 |  |  |  | 25 | 325 | 248,283 |  |
| A10155 |  |  |  | 3 | 296 | 1,964,489 |  |
| A10156 |  |  |  | 11 | 286 | 1,514,170 |  |
| A10157 |  |  |  | 10 | 280 | 619,433 | 1 |
| A10158 |  |  |  | 21 | 231 | 81,835 | 6 |
| A10159 |  |  |  | 7 | 335 | 522,038 |  |
| A10160 |  |  |  | 16 | 335 | 82,389 | 1 |
| A10161 |  |  |  | 18 | 288 | 955,697 |  |
| A10162 |  |  |  | 14 | 310 | 780,587 |  |
| A10163 |  |  |  | 20 | 293 | 997,220 |  |
| A10164 |  |  |  | 6 | 309 | 661,538 |  |
| A10165 |  |  |  | 12 | 278 | 90,936 | 2 |
| A10166 |  |  |  | 7 | 285 | 260,253 |  |
| A10167 |  |  |  | 4 | 314 | 1,706,159 |  |
| A10168 | 6 | 6 |  | 9 | 339 | 690,750 | 1 |
| A10169 |  |  |  |  | 344 | 906,745 | 0.01 |
| A10170 | 4 | 9 | 1 | 3 | 308 | 208,130 | 1 |
| A10171 |  |  |  | 8 | 263 | 126,878 | 2 |
| A10172 |  |  |  | 7 | 326 | 611,925 |  |
| A10173 |  |  |  | 17 | 258 | 208,453 | 2 |
| A10174 |  |  |  | 13 | 287 | 342,318 | 2 |
| A10175 |  |  |  | 3 | 343 | 448,871 |  |
| A10176 |  |  |  | 10 | 332 | 489,647 |  |
