# Supplementary material for: High-Resolution Coproecology: Using Coprolites to Reconstruct the Habits and Habitats of New Zealand’s Extinct Upland Moa (Megalapteryx didinus)
Source: PLoS One. 2012 Jun 29;7(6):e40025. doi: 10.1371/journal.pone.0040025 (PMC3386916; doi:10.1371/journal.pone.0040025)
Supplement: Table S2 — Plant macrofossils and invertebrate remains from Euphrates Cave moa coprolites. (DOCX) [file pone.0040025.s007.docx]

| Sample | A101## | 42 | 43 | 44 | 45 | 46 | 47 | 48 | 49 | 50 | 51 | 52 | 53 | 54 | 55 | 56 | 57 | 58 | 59 | 60 | 61 | 62 | 63 | 64 | 65 | 66 | 67 | 68 | 69 | 70 | 71 | 72 | 73 | 74 | 75 | 76 |
| --- | --- | --- | --- | --- | --- | --- | --- | --- | --- | --- | --- | --- | --- | --- | --- | --- | --- | --- | --- | --- | --- | --- | --- | --- | --- | --- | --- | --- | --- | --- | --- | --- | --- | --- | --- | --- |
|  | Subsample mass (g) | 9.0 | 2.0 | 4.2 | 6.0 | 3.3 | 9.1 | 4.4 | 8.3 | 4.9 | 5.0 | 5.5 | 5.3 | 5.4 | 9.6 | 2.3 | 8.7 | 3.7 | 4.7 | 1.5 | 3.4 | 12.1 | 1.9 | 8.1 | 12.3 | 3.4 | 4.1 | 4.7 | 3.6 | 3.3 | 0.7 | 8.8 | 3.5 | 6.6 | 2.0 | 2.9 |
| Taxa | Material |  |  |  |  |  |  |  |  |  |  |  |  |  |  |  |  |  |  |  |  |  |  |  |  |  |  |  |  |  |  |  |  |  |  |  |
| *Nothofagus menziesii* | Leaf fragments |  |  |  |  |  | ✓ |  | ✓ |  |  |  |  |  |  |  |  |  |  | ✓ |  |  |  |  | ✓ | ✓ |  |  |  |  |  |  |  |  |  | ✓ |
| *Fuchsia excorticata* | Seed |  |  |  |  |  |  |  |  |  |  |  |  |  |  |  |  |  | 21 |  |  |  |  |  |  |  |  |  |  | 5 |  |  |  |  |  |  |
| Epacridaceae | Leaf |  |  |  |  |  |  |  | 1 |  |  |  |  |  |  |  |  |  |  |  |  |  |  |  |  |  |  |  |  |  |  |  |  |  |  |  |
| *Gaultheria* sp. | Seed |  |  |  |  | 1 |  |  |  | 18 | 1 |  |  |  | 1 |  | 1 |  | 2 |  |  |  |  |  | 1 |  |  | 11 |  | 12 |  |  |  | 3 |  |  |
| Lobeliaceae (cf. *Pratia*) | Seed |  |  | 9 |  |  |  |  |  |  |  |  |  |  |  |  |  |  |  | 6 |  |  |  |  |  |  |  | 6 |  | 5 |  |  |  |  |  |  |
| *Ranunculus* sp. | Seed |  |  | 7 |  |  | 2 |  |  | 1 |  |  |  | 1 |  |  |  |  | 4 |  |  | 2 |  |  |  | 2 |  |  |  |  |  | 7 |  | 2 |  | 5 |
| *Colobanthus* sp. | Seed |  |  |  |  |  |  |  |  |  |  |  |  |  |  |  |  |  | 3 |  |  |  |  |  |  |  |  |  |  |  |  |  |  | 1 |  |  |
| *Urtica* sp.? | Seed |  |  | 1 |  |  | 2 |  |  |  |  |  |  |  |  |  |  |  | 1 | 7 |  |  |  |  |  |  |  |  |  |  | 2 |  |  |  |  |  |
| Poaceae | Florets |  |  | 1 |  |  |  |  |  |  |  |  |  |  |  |  |  |  |  | 3 |  |  |  |  |  |  |  |  |  |  |  |  |  |  | 1 |  |
| Poaceae | Seed |  |  |  |  |  |  |  |  |  |  |  |  |  |  |  |  |  |  |  |  |  |  |  |  |  |  |  |  | 1 |  |  |  |  |  |  |
| *Cf. Scirpus* sp. | Seed |  |  |  |  |  | 9 |  | 1 |  |  |  | 1 | 6 |  |  | 1 |  | 9 |  |  |  | 3 |  |  | 1 | 5 |  |  |  |  | 5 |  | 4 | 3 | 7 |
| *Cf. Carex* sp. | Seed |  |  |  |  |  |  |  |  | 1 |  |  |  |  | 1 |  |  |  | 5 |  |  |  |  |  |  |  |  |  |  |  |  |  |  |  |  |  |
| Fern | Leaf fragments |  |  | ✓ |  |  |  |  | ✓ |  |  |  |  |  |  |  |  |  |  | ✓ |  |  |  |  |  |  |  |  |  |  |  |  |  |  |  |  |
| Bryophyta | Fragments |  | ✓ | ✓ |  |  |  | ✓ | ✓ | ✓ |  |  |  | ✓ |  |  | ✓ |  | ✓ |  |  | ✓ | ✓ |  |  | ✓ |  |  | ✓ | ✓ | ✓ |  | ✓ |  | ✓ | ✓ |
| Unidentified | Seed/seed fragments |  |  | 2 | 1 |  | 1 | 2 |  | 1 |  |  |  |  | 1 |  |  |  | 4 | 4 |  |  |  | 1 |  | 1 |  |  |  |  |  | 2 |  | 4 | 4 |  |
| Unidentified | Leaf/leaf fragments |  |  |  |  |  |  |  |  |  | ✓ |  |  |  |  |  |  |  |  | ✓ |  |  |  |  |  |  | ✓ |  |  |  |  |  |  |  |  |  |
|  |  |  |  |  |  |  |  |  |  |  |  |  |  |  |  |  |  |  |  |  |  |  |  |  |  |  |  |  |  |  |  |  |  |  |  |  |
| Invertebrate | Fragments |  |  | ✓ |  |  | ✓ |  |  | ✓ | ✓ |  |  | ✓ | ✓ |  | ✓ | ✓ |  | ✓ | ✓ | ✓ |  |  | ✓ |  |  |  | ✓ |  |  | ✓ |  |  |  | ✓ |
| Invertebrate (Ptiliidae?) | Fragments |  |  | ✓ |  |  |  |  |  |  |  |  |  |  |  |  |  |  |  |  |  |  |  |  |  |  |  |  |  |  |  |  |  |  |  |  |
|  |  |  |  |  |  |  |  |  |  |  |  |  |  |  |  |  |  |  |  |  |  |  |  |  |  |  |  |  |  |  |  |  |  |  |  |  |
|  |  |  |  |  |  |  |  |  |  |  |  |  |  |  |  |  |  |  |  |  |  |  |  |  |  |  |  |  |  |  |  |  |  |  |  |  |
|  |  |  |  |  |  |  |  |  |  |  |  |  |  |  |  |  |  |  |  |  |  |  |  |  |  |  |  |  |  |  |  |  |  |  |  |  |
